# Test-Retest Reliability of Graph Metrics in Functional Brain Networks: A Resting-State fNIRS Study

**DOI:** 10.1371/journal.pone.0072425

**Published:** 2013-09-09

**Authors:** Haijing Niu, Zhen Li, Xuhong Liao, Jinhui Wang, Tengda Zhao, Ni Shu, Xiaohu Zhao, Yong He

**Affiliations:** 1 State Key Laboratory of Cognitive Neuroscience and Learning, Beijing Normal University, Beijing, China; 2 Center for Cognition and Brain Disorders, Hangzhou Normal University, Hangzhou, China; 3 Zhejiang Key Laboratory for Research in Assessment of Cognitive Impairments, Hangzhou, China; 4 Imaging Department, Shanghai TongJi Hospital, TongJi University, Shanghai, China; Indiana University, United States of America

## Abstract

Recent research has demonstrated the feasibility of combining functional near-infrared spectroscopy (fNIRS) and graph theory approaches to explore the topological attributes of human brain networks. However, the test-retest (TRT) reliability of the application of graph metrics to these networks remains to be elucidated. Here, we used resting-state fNIRS and a graph-theoretical approach to systematically address TRT reliability as it applies to various features of human brain networks, including functional connectivity, global network metrics and regional nodal centrality metrics. Eighteen subjects participated in two resting-state fNIRS scan sessions held ∼20 min apart. Functional brain networks were constructed for each subject by computing temporal correlations on three types of hemoglobin concentration information (HbO, HbR, and HbT). This was followed by a graph-theoretical analysis, and then an intraclass correlation coefficient (*ICC*) was further applied to quantify the TRT reliability of each network metric. We observed that a large proportion of resting-state functional connections (∼90%) exhibited good reliability (0.6< *ICC* <0.74). For global and nodal measures, reliability was generally threshold-sensitive and varied among both network metrics and hemoglobin concentration signals. Specifically, the majority of global metrics exhibited fair to excellent reliability, with notably higher *ICC* values for the clustering coefficient (HbO: 0.76; HbR: 0.78; HbT: 0.53) and global efficiency (HbO: 0.76; HbR: 0.70; HbT: 0.78). Similarly, both nodal degree and efficiency measures also showed fair to excellent reliability across nodes (degree: 0.52∼0.84; efficiency: 0.50∼0.84); reliability was concordant across HbO, HbR and HbT and was significantly higher than that of nodal betweenness (0.28∼0.68). Together, our results suggest that most graph-theoretical network metrics derived from fNIRS are TRT reliable and can be used effectively for brain network research. This study also provides important guidance on the choice of network metrics of interest for future applied research in developmental and clinical neuroscience.

## Introduction

The human brain is a highly complex system that can be represented as a structurally or functionally interconnected network that assures rapid segregation and integration of information processing. Considerable progress has recently been made in describing the topological organization of human whole-brain networks using neuroimaging data and graph-theoretical approaches [1], [2], [3], [4]. A wealth of research has demonstrated that human structural or functional brain networks can be constructed using a variety of non-invasive neuroimaging techniques, including structural MRI [5], diffusion MRI [6], [7], [8], blood oxygenation level dependent functional MRI (BOLD-fMRI) [9], [10], [11], and functional near-infrared spectroscopy (fNIRS) [12]. Graph theory analysis has further revealed that these brain networks exhibit many non-random topological properties, such as small-world efficiency, modularity and highly connected hubs. These quantitative topological properties have been found to change with normal development, aging and disease conditions (for reviews, see [10], [13], [14], [15], [16]).

Compared to traditional functional imaging techniques such as BOLD-fMRI, fNIRS is a newly developed but promising neuroimaging technology that uses light in the near-infrared spectrum (670–900 nm) to noninvasively monitor cortical hemodynamic variations induced by neural activation [17], [18]. The primary advantages of fNIRS over other neuroimaging techniques include portability, effective cost, ecological validity and higher temporal resolution. One of the main contributions of fNIRS research is the detection of resting-state functional connectivity (RSFC), which chara**c**terizes the synchronization of spontaneous neural activity between spatially remote brain regions [19], [20], [21], [22], [23], [24], [25]. Very recently, we proposed resting-state fNIRS (R-fNIRS) and graph-theoretical approaches to explore the topological architecture of human whole-brain functional networks [12]. In the R-fNIRS based network analysis framework, the channels are considered vertices, and RSFCs between channels are considered edges. Using this approach, we previously demonstrated that R-fNIRS can effectively characterize the topological attributes of human brain networks, such as small-world efficiency, modularity and highly connected hubs [12]. The results of this study were also consistent with recent findings from BOLD-fMRI research, suggesting the feasibility and validity of combining R-fNIRS and graph theory analysis to identify the functional properties of human brain networks. However, it still remains largely unknown whether the topological brain network measures derived from R-fNIRS data are repeatable or test-retest (TRT) reliable. If these metrics can be consistently replicated, they have the potential to be adopted as biomarkers in further cognitive neuroscience and clinical research. This could prove important in longitudinal studies of brain changes and in research using repeated measurements in the context of normal development or even pharmacological treatment. Consequently, it is an important and necessary task to explore the TRT reliability of functional brain networks derived from human R-fNIRS data.

In the present study, we aimed to provide a comprehensive assessment of the TRT reliability of graph metrics for R-fNIRS brain networks. To this end, we collected human R-fNIRS data in a group of healthy young adults over two sessions with a 20-minute interval between sessions. For each participant, we constructed whole-brain functional networks by computing temporal correlations between the time series of pairs of channels based on hemoglobin concentration. We further analyzed three sets of network properties (functional connectivity, global network metrics and regional nodal metrics) using graph-theoretical approaches [12]. Finally, intraclass correlation coefficients (*ICC*) were calculated to assess the TRT reliability of these network metrics.

## Materials and Methods

### Subjects and protocol

Twenty-one healthy right-handed subjects (mean age: 24.5 years; range: 21–27 years; 17 male) participated in this study. All subjects were college students recruited from Beijing Normal University. The study was approved by the Review Board at the State Key Laboratory of Cognitive Neuroscience and Learning, Beijing Normal University, and written informed consent was obtained from each subject prior to the experiment. Each participant underwent two eleven-minute sessions of resting-state fNIRS scanning separated by an interval of twenty minutes. Participants were instructed to remain still during scanning and to keep their eyes closed without falling asleep. During the rest interval, participants were instructed to sit still while wearing the probe holder, but they were allowed slight body and head motion. The signal quality from all measurement channels will be separately checked before the performance of the second session for each participant. If the signal at one or several channel(s) showed low signal-to-noise ratio, the whole probe holder or the specific source-detector pair determining the defective measurement channel will be readjusted.

### Data acquisition and preprocessing

**Figure 1:**
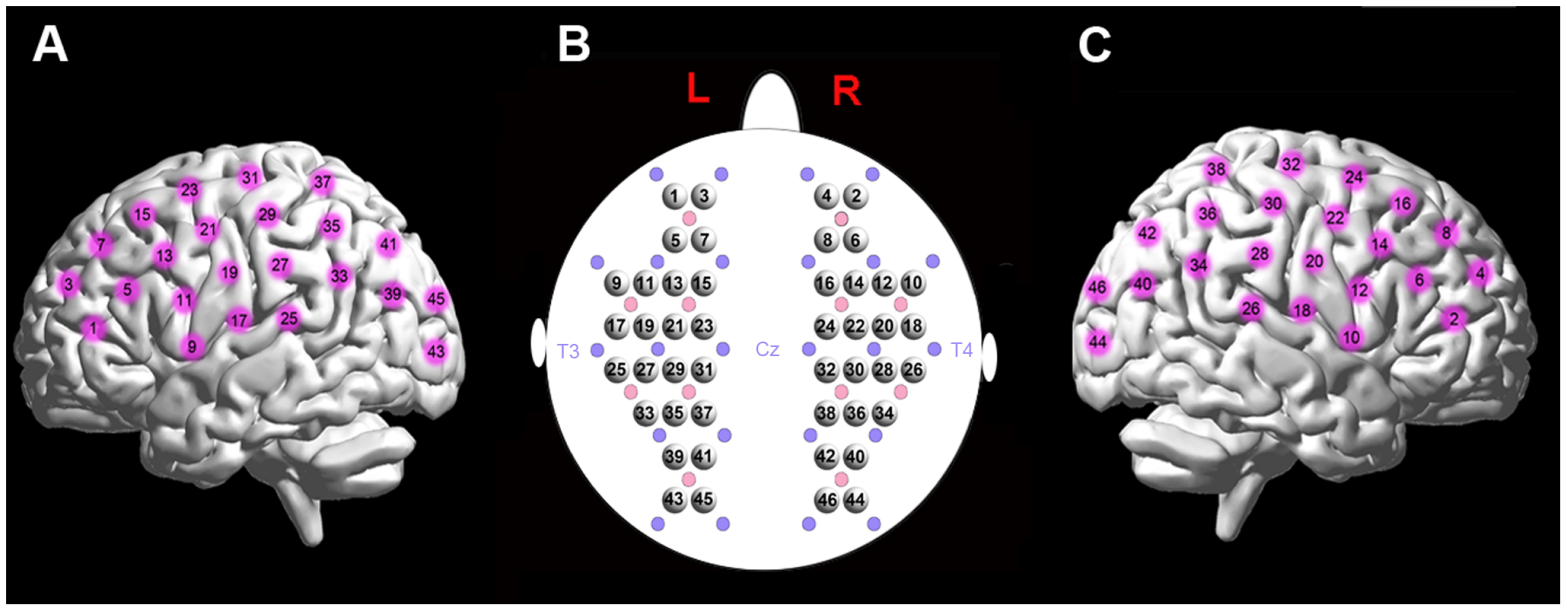
Schematic of fNIRS channel localization. (A) and (C) display the left and right view of measurement channels on an anatomical brain image. (B) The arrangement of the whole-head 46 measurement channels on a brain template. Note that the red and purple solid circles represent sources and detectors, respectively.

The data were acquired using a continuous wave (CW) near-infrared optical imaging system (CW6, TechEn Inc., MA, USA) equipped with 12 laser sources and 24 detectors designed to cover almost the entire head, including the frontal, temporal, parietal, and occipital lobes. The probe arrays allowed for 46 different measurement channels, with 3.2 cm of source-detector separation (Fig. 1). The positioning of the probes was based on the international 10–20 system, and the positions were measured at each scanning session to establish consistent placement. Specifically, two detectors around channels 17 and 18 were placed in T3 and T4, and the midpoint of the two detectors was localized in Cz. The system generated two wavelengths of near-infrared light (690 and 830 nm) and measured changes in the concentrations of oxyhemoglobin (HbO) and deoxyhemoglobin (HbR) based on the modified Beer-Lambert law [26]. The sum of the HbO and HbR signals was defined as the total hemoglobin (HbT) concentration. Considering that the spontaneous fluctuations of each concentration data usually include various non-neural signals (e.g., respiratory and cardiac noises), we thus adopted a band-pass filtering with cutoff frequencies of 0.009 and 0.08 Hz to reduce the effects of these noises [23], [27], [28], which was similar to previous resting-state fMRI study [29]. Three subjects were excluded due to poor contact between the probe and scalp, leaving 18 subjects on which further analysis was performed. To obtain a stable signal, the first minute of each hemodynamic time series was discarded. The sampling rate for the optical signal was set to 25 Hz, which resulted in 15,000 sample points for each 10-min dataset for each subject.

### Network construction

In a previous study, we developed a procedure to construct whole-brain functional networks based on R-fNIRS data [12]. Briefly, RSFC was calculated with Pearson correlation analysis between the time series of every pair of nodes, where nodes were measurement channels. This resulted in a {46×46} correlation matrix for each participant at each session. The correlation matrix was then thresholded into a binary matrix that described the topological organization of the functional networks. As in our previous study [12], we chose sparsity (*S*) as a threshold measure, and sparsity was defined as the number of actual connections divided by the maximum possible number of connections in the network, ranging from 0.01 to 0.99 at an interval of 0.01.

### Network analysis

We explored two sets of topological measures for the brain networks: 1) global network metrics: small-world properties (clustering coefficient *C_p_*, characteristic path length *L_p_*, normalized clustering coefficient *γ*, normalized characteristic path length *λ,* and small-world σ), efficiency parameters (local efficiency *E_loc_* and global efficiency *E_glob_*), modularity *Q*, hierarchy *β*, and assortativity *r*; and 2) nodal characteristics: nodal degree, nodal efficiency, and nodal betweenness. For each subject at each sparsity level, we computed the entire network metrics based on an in-house GRETNA package. These metrics are described in further detail below.

#### Small-world

Small-world measures of a network (clustering coefficient, *C_p_*, and characteristic path length, *L_p_*) were originally proposed by Watts and Strogatz (1998). Briefly, *C_p_* is the average of clustering coefficients over all nodes in a network, where the clustering coefficient of a node *v* is defined as the number of existing connections among the neighbors of the node divided by all their possible connections. *C_p_* quantifies the extent of local cliquishness of a network [30], [31]. *L_p_* is defined as the average of the shortest path lengths (i.e., the minimum number of edges that link any two nodes of the network) between any pair of nodes in the network. It quantifies the capability of parallel information propagation within a network [32]. Mathematically, a real network would be considered small-world if it meets the following requirements: *γ*  =  *C_p_^rea^* ^l^/*C_p_^rand^* > 1 and *λ*  =  *L_p_^rea^* ^l^/*L_p_^rand^* 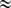 1 [31], where *C_p_^rand^* and *L_p_^rand^* are the mean clustering coefficient and characteristic path length of matched random networks that preserve the same number of nodes, edges, and degree distribution as the real network [33]. The two metrics can also be summarized into a simple quantitative small-worldness index *σ*: *σ*  =  *γ*/*λ* >1 [34].

#### Efficiency

The global efficiency of a network is defined as the average inverse shortest path length. Correspondingly, local efficiency is defined as the efficiency of the local sub-group of a node *v* that comprises only the direct neighbors of node *v*. Global and local efficiency measure the ability of a network to transmit information at the global and local level, respectively [32].

#### Modularity

The modularity measure *Q* is defined by the following equation [35]:
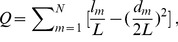
(1)where *N* is the number of modules, *L* is the total number of links in the network, *l_m_* is the total number of links between nodes in module *m*, and *d_m_* is the sum of the degrees of the nodes in module *m*. It has been shown that connections are usually denser within modules than between them.

#### Hierarchy

In a hierarchical network, nodes with a high degree show a lower clustering coefficient, while nodes with a low degree show a higher clustering coefficient, reflecting the top-down organization of the network. This ratio can be quantified by [36]:
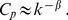
(2)


The coefficient *β* was estimated by fitting a linear regression line to the plot of *log*(*C_p_*) versus *log*(*k*).

#### Assortativity

Assortativity is a measure of how often nodes are linked to other nodes with a similar degree. Accordingly, it is defined as the correlation between the degree of a node and the mean degree of its direct neighbors [37].

#### Nodal degree, efficiency, and betweenness

The degree of a node *v* is defined as the number of edges linked directly to the node. The efficiency of a node *v* is the inverse of the harmonic mean of the minimum path length between the node *v* and all other nodes in the network [30], [38]. Nodal efficiency is a measure of local connectivity. The betweenness of a node *v* is defined as the number of shortest paths between any two nodes that run through node *v* [39]. Nodal betweenness quantifies how important a node is within a network. It is defined as:
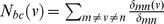
(3)where *δ_mn_* is the total number of shortest paths from node *m* to node *n*, and*δ_mn_*(*v*) is the number of shortest paths from node *m* to node *n* that pass through node *v*. A node with a high *N_bc_* indicates a greater impact of the node on information flow across the whole network.

### Test-retest reliability analysis

**Figure 2:**
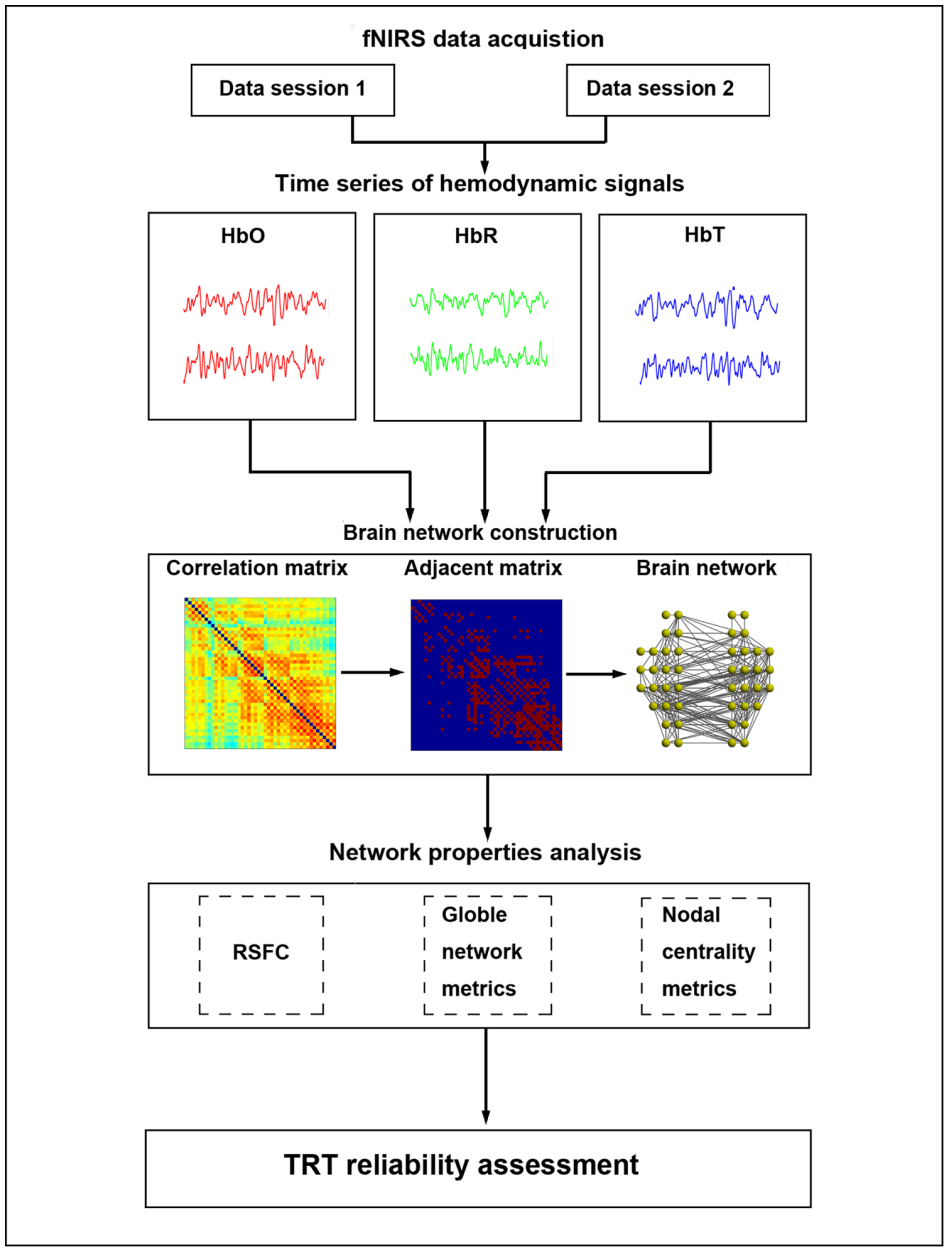
Demonstration of R-fNIRS brain network reliability analysis.

The intraclass correlation coefficient was used to evaluate the test–retest reliability of R-fNIRS brain networks [40]. To comprehensively investigate the test–retest reliability of the graph theory-derived R-fNIRS network, we analyzed the reliability of R-fNIRS network properties from three perspectives: resting-state functional connectivity (RSFC), global network metrics and regional nodal centrality metrics. For each metric, the individual RSFC or network parameters values of all subjects in both two sessions were first merged into one 18×2 matrix, with rows corresponding to participants and columns corresponding to repeated measurements in two sessions. Notably, for RSFC, the 18×2 matrix represents the connection strength between any two nodes across two sessions; for network metrics, each 18×2 matrix represents the global or nodal parameter values across two sessions. Using a one-way ANOVA on each matrix, with random participant effects, we split the total variance of each network property into between-participant (*MS_b_*) and within-participant (*MS_w_*) variance. Then, the *ICC* values were calculated according to the following equation [40]:
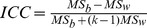
(4)where *k* is the number of repeated observations per participant, *MS_b_* is the between-participant variance and *MS_w_* is the within-participant variance. All reliability indices discussed above were evaluated according to the criteria proposed by Sampat and Winer et al. [41], [42], where an *ICC* value from 0 to 0.25 indicates poor reliability; 0.25 to 0.4 low reliability; 0.4 to 0.6 fair reliability; 0.6 to 0.75 good reliability; and 0.75 to 1.0 excellent reliability. Notably, for one metric, if the within-participant variance is larger than the between-participant variance, it will lead to a negative *ICC*. Theoretically, such a situation is impossible [43], and the reasons for negative values is even currently unclear [44]. Thus, the negative *ICC* will be set to zero (not reliable), as generally suggested [24], [45]. Because we constructed the network over a continuous sparsity threshold range, the *ICC* value is a function of the threshold. To further conduct a threshold-independent reliability assessment, we also calculated the integral or area under the curve (AUC) of each network metric [46] to be used as the corresponding *ICC* scalar of network evaluation. Figure 2 displays a flowchart of the reliability analysis of the R-fNIRS brain network.

### Denoising using independent component analysis

A general problem with R-fNIRS recordings is that the acquired signals often include various different types of signal noise and artifacts (e.g., instrumental noise, motion-induced artifacts and physiological noises). Independent component analysis (ICA) provides an important technique to reduce the effects of these noise sources [25], [47], [48]. In this study we re-analyzed our R-fNIRS data and assessed the TRT reliability of functional brain networks after denoising based on a temporal ICA analysis. The analysis procedure we used was similar to that in previous R-fNIRS studies [24], [25] and was performed here by using a publicly available software, *FastICA v2.5* (www.cis.hut.fi/projects/ica/fastica/). Specifically, the parameter settings of the *FastICA* algorithm [49] included: number of iteration steps  = 10000, step length  = 0.00001, approach  = “deflation”, initial value  = “random”, nonlinearity  =  “skew”, fine-tune  =  “on”, and stabilization  =  “on”. The ICA analysis was separately performed on the raw data of HbO, HbR and HbT with the following procedures: extracting steady hemoglobin concentration signals (e.g. removing the initial time points from total data length), reducing the dimensionality of the data with principal component analysis (PCA), performing ICA analysis on the reduced dimensional data, identifying noise components, removing noise from the measured data, and calculating “real” neural activity signals. *FastICA* can automatically adjust the results of the analysis required to compensate for these preprocessing steps. The number of retained principal components (PCs) was determined according to the minimum number of PCs that retained more than 99% of data variance [50]. Meanwhile, the number of independent components through ICA decomposition was set to be equal to the number of PCs. After ICA analysis, the components related to noise and artifacts were identified from three aspects: temporal profiles, spatial maps and power spectra. A component would be considered noise if it met one of the following conditions [25]: *i*) the corresponding temporal profile included sudden jumps, slowly-varied U or inverted U-shaped spike, or numerous inter-current quick spikes; *ii*) the dominant frequency of power spectra of the component was outside the range of 0.01∼0.1Hz; *iii*) the spatial map of the component presented a global and spatially dispersive pattern. Notably, the map could be approximately related to the interference in superficial layers of head [48]. After identifying these types of noise components, each concentration signal of HbO, HbR, and HbT was reconstructed with a particular component eliminated from the original R-fNIRS time course by replacing zero in the corresponding column of mixing matrix [48]. Finally, we reconstructed the brain networks and then computed their topological properties and the *ICCs* as described above.

## Results

### TRT reliability of RSFC maps

**Figure 3:**
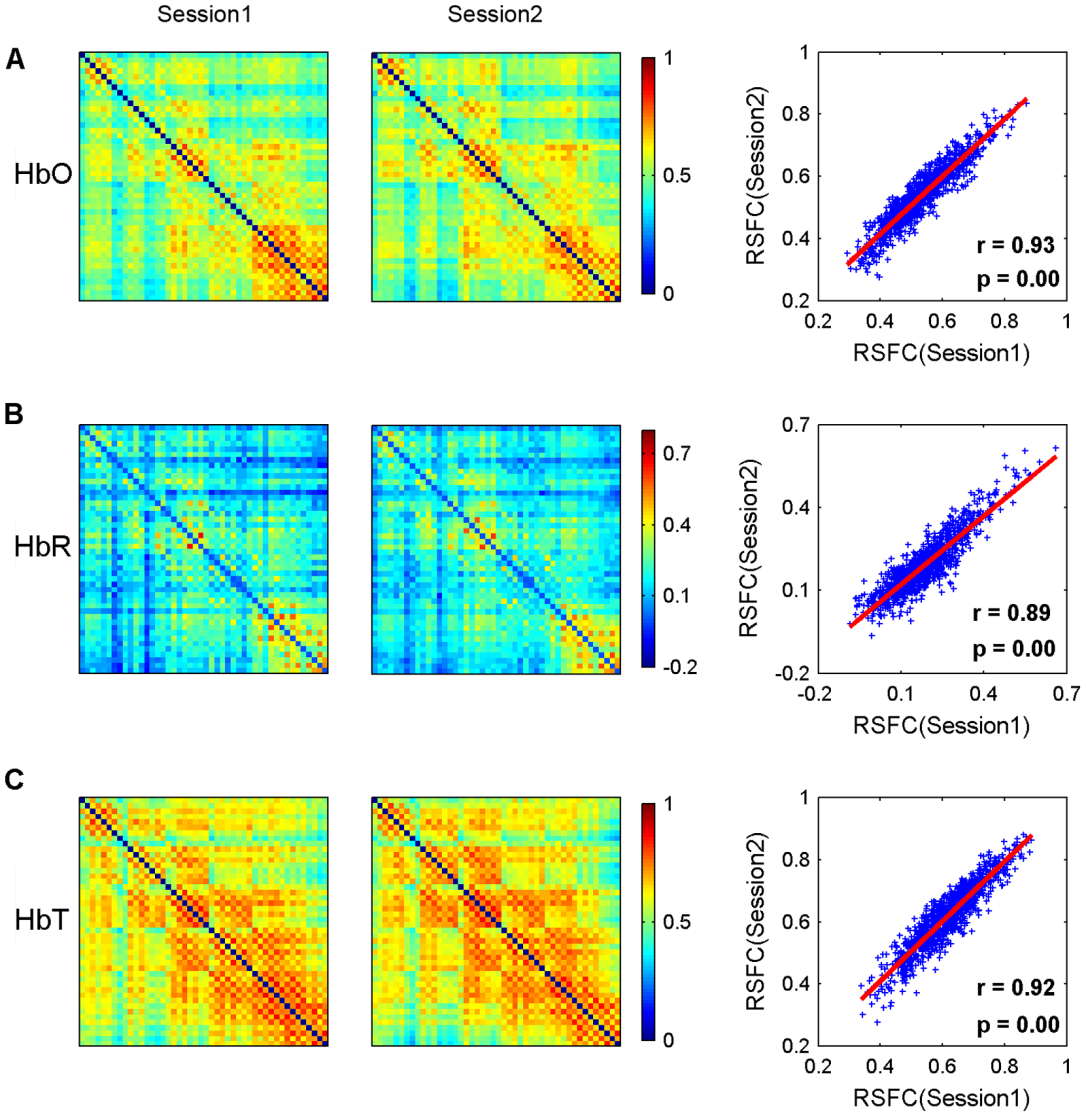
Spatial similarity of RSFC maps. Group-level RSFC maps for session 1 and session 2 and their Pearson correlation are displayed in the first to third columns. Figures (A) to (C) correspond to the RSFC data derived from HbO, HbR, and HbT, respectively. High similarity between sessions was observed in both the qualitative visual inspection and quantitative correlational analysis.

The group-level RSFC maps of two R-fNIRS scans (session 1 and session 2) and the linear relationship between them are presented in Fig. 3A–C. In general, the spatial patterns of the RSFC maps exhibited high similarity between sessions across HbO, HbR, and HbT, and this can be observed in both the qualitative visual inspection and quantitative correlation analysis (HbO: *r* = 0.93, *p*<0.0001; HbR: *r* = 0.89, *p*<0.0001; HbT: *r* = 0.92, *p*<0.0001). The individual RSFC correlation between sessions was also examined independently, and these results are summarized in Table 1. The RSFC maps between sessions also manifested high similarities (*p*<0.001) for each participant and each hemoglobin concentration signal; the average correlation coefficients across subjects were 0.70±0.09 (mean and standard deviation) for HbO, 0.67±0.13 for HbR, and 0.69±0.10 for HbT.

**Table 1:** Pearson correlations at individual-level RSFC maps between sessions.

Subject	HbO		HbR		HbT	
ID	*r*	*p*	*r*	*p*	*r*	*p*
**1**	0.64	2.64×10^−121^	0.40	2.34×10^−41^	0.76	3.60×10^−192^
**2**	0.66	2.69×10^−129^	0.62	5.54×10^−110^	0.66	5.67×10^−132^
**3**	0.56	3.83×10^−86^	0.59	1.81×10^−96^	0.56	2.83×10^−88^
**4**	0.75	1.49×10^−184^	0.63	1.16×10^−117^	0.66	1.04×10^−130^
**5**	0.76	2.72×10^−198^	0.69	3.22×10^−145^	0.60	8.93×10^−104^
**6**	0.75	7.12×10^−188^	0.78	1.12×10^−210^	0.74	6.87×10^−183^
**7**	0.54	3.86×10^−79^	0.77	1.87×10^−206^	0.72	7.23×10^−263^
**8**	0.76	6.81×10^−198^	0.53	3.35×10^−75^	0.79	9.78×10^−221^
**9**	0.85	4.04×10^−294^	0.88	0. 00	0.74	3.36×10^−182^
**10**	0.82	1.30×10^−253^	0.78	3.31×10^−208^	0.90	0.00
**11**	0.69	1.64×10^−147^	0.61	1.86×10^−106^	0.74	1.40×10^−176^
**12**	0.66	1.51×10^−132^	0.63	2.81×10^−117^	0.62	1.15×10^−109^
**13**	0.69	9.30×10^−147^	0.52	1.08×10^−72^	0.52	9.43×10^−72^
**14**	0.73	2.17×10^−173^	0.75	3.82×10^−186^	0.72	8.98×10^−166^
**15**	0.57	1.01×10^−88^	0.55	1.33×10^−81^	0.55	1.84×10^−84^
**16**	0.65	5.15×10^−127^	0.78	1.41×10^−214^	0.75	7.91×10^−189^
**17**	0.86	4.53×10^−297^	0.90	0. 00	0.81	1.64×10^−238^
**18**	0.67	2.42×10^−137^	0.56	4.73×10^−88^	0.66	1.86×10^−128^
**mean**	**0.70**		**0.67**		**0.69**	
**std**	**0.09**		**0.13**		**0.10**	

**Figure 4:**
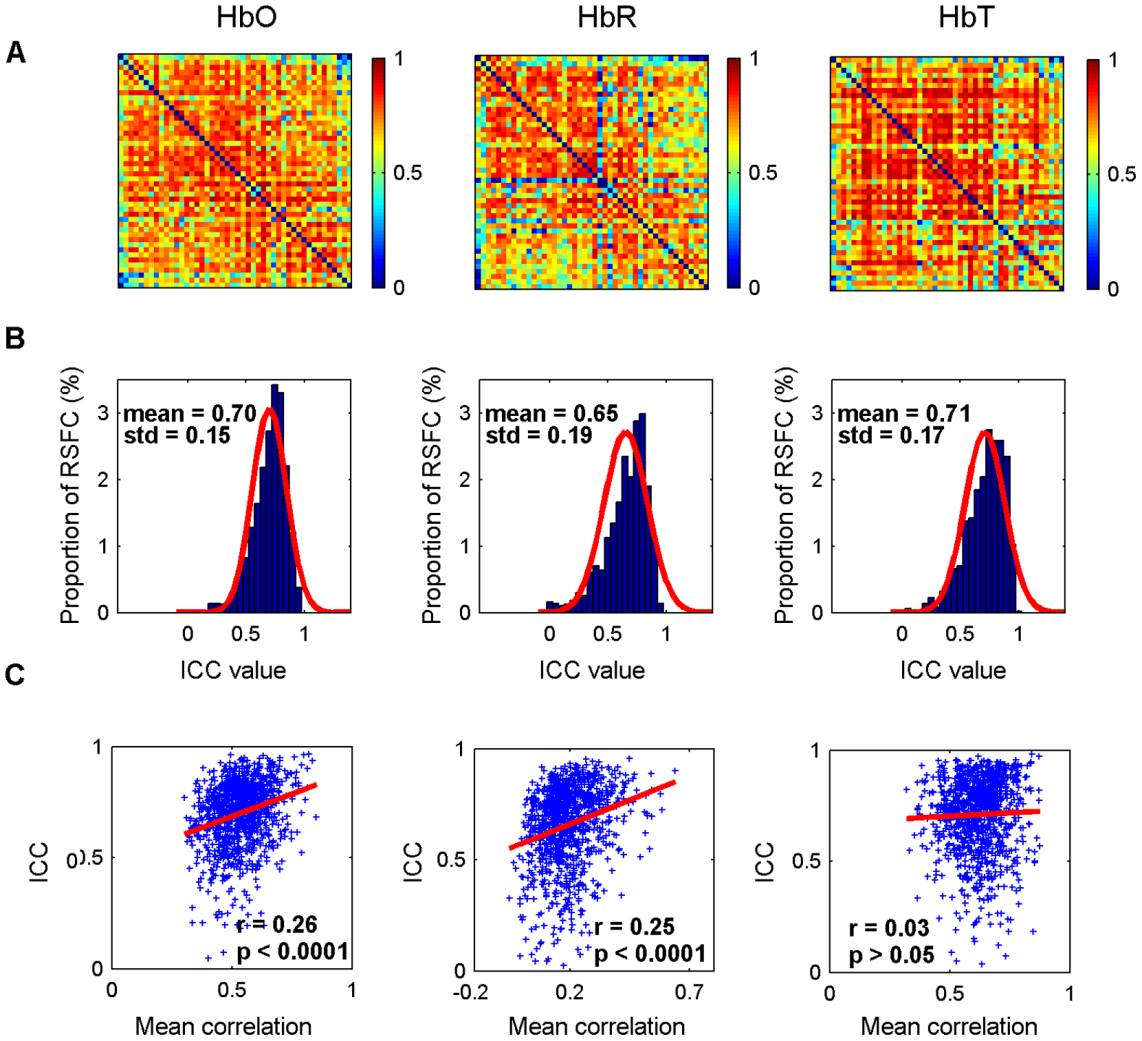
Reliability analysis of RSFC maps. The first to third columns correspond to the data derived from HbO, HbR, and HbT, respectively. (A, B) The TRT reliability of RSFC maps and their corresponding reliability distributions. The reliability displays approximately normal configuration for all 1035 (i.e., 46×45/2) connections. The connections exhibit good reliability across HbO (mean *ICC* values 0.70), HbR (0.65) and HbT (0.71). (C) The relationship between RSFC strength and reliability as assessed by scatterplots. Each dot represents the group-level RSFC strength and the corresponding *ICC* value at the same connections. The trend lines were obtained by a linear least-squares fit method. Significant (*p*<0.05) positive correlations were found for HbO and HbR signals, suggesting stronger RSFC leads to higher reliability for both these signals.

The consistency of these measures was further confirmed by an analysis of the reliability of the RSFC maps. Figure 4 shows the TRT reliability for the RSFC maps and their corresponding reliability distributions. The average *ICC* values across all 1035 (i.e., 46×45/2) connections were 0.70±0.15 for HbO, 0.65±0.19 for HbR, and 0.71±0.17 for HbT. These *ICC* values also displayed an approximately normal distribution for all 1035 connections at each concentration signal (Fig. 4B). Notably, most of the connections for each concentration signal also exhibited fair to excellent reliability. For HbO, 985 of 1035 (95%) functional connections exhibited fair to excellent reliability (fair: 214, 20.7%; good: 402, 38.8%; excellent: 369, 35.7%). Similarly, for HbR and HbT, 902 of 1035 (87%) and 948 of 1035 (92%) functional connections exhibited fair to excellent reliability (HbR: fair: 244, 23.6.7%; good: 333, 32.2%; excellent: 328, 31.7%. HbT: fair: 229, 22.1%; good: 321, 31%; excellent: 398, 38.5%). Furthermore, a Pearson's correlation analysis revealed significant positive correlations between RSFC strength and reliability for HbO (*r* = 0.26, *p*<0.0001) and HbR (*r* = 0.25, *p*<0.0001) signals, whereas no significant correlations were found for HbT (*r* = 0.03, *p*>0.05) (Fig. 4C).

### TRT reliability of global network metrics

**Figure 5:**
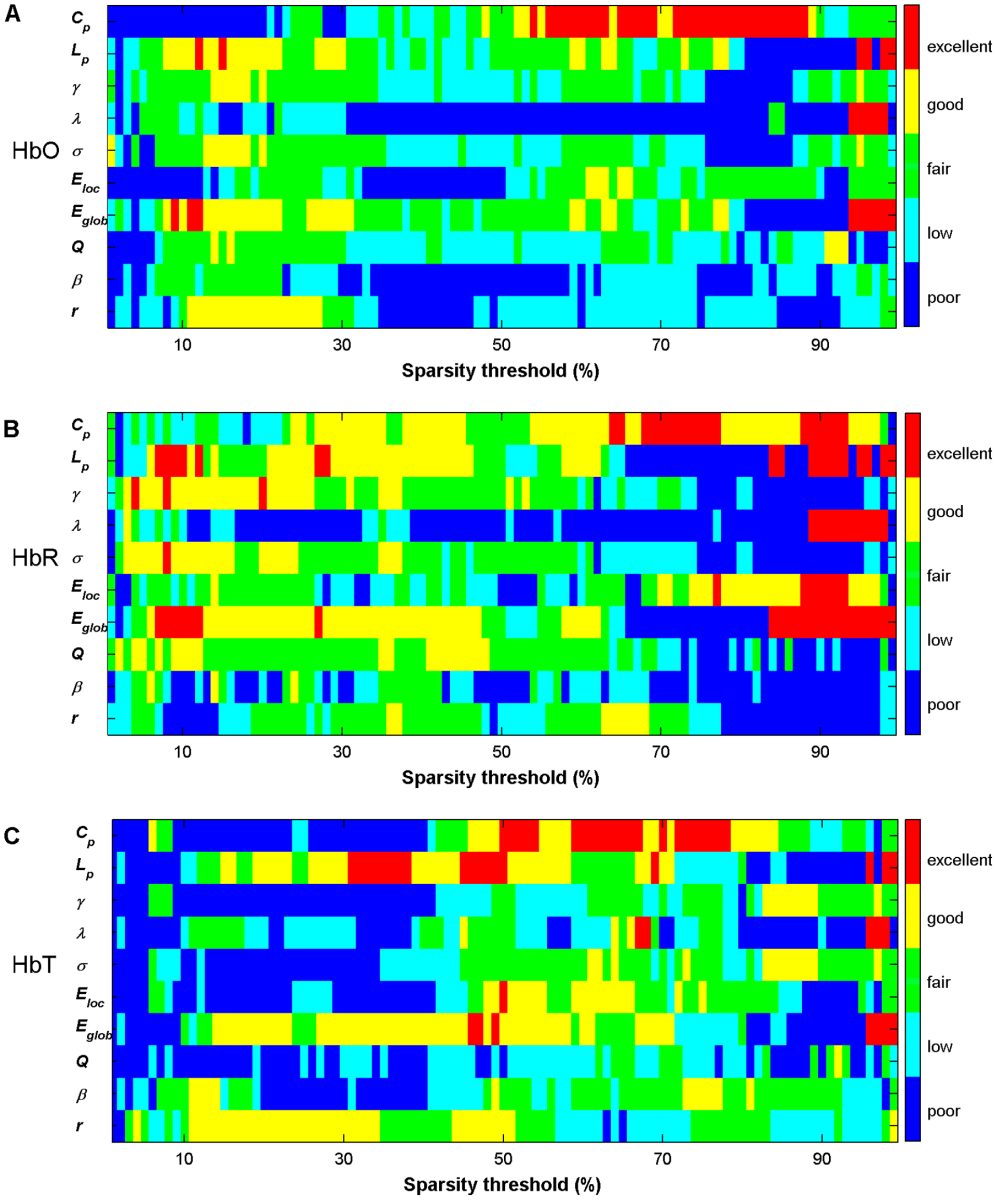
TRT reliability of global network metrics as a function of sparsity threshold. (A–C) The global metric reliability was derived from HbO, HbR, and HbT, respectively. Five colors correspond to five different reliability grades. The red, yellow, green, cyan, and blue colors represent excellent (0.75< *ICC* <1), good (0.6< *ICC* <0.75), fair (0.4< *ICC* <0.6), low (0.25< *ICC* <0.4), and poor (*ICC*<0.25) reliability of global network metrics, respectively. *C_p_*, *L_p_*, *γ*, *λ,* and σ denote the clustering coefficient, characteristic path length, normalized clustering coefficient, normalized characteristic path length, and small-world, respectively. *E_loc_* and *E_glob_* denote local efficiency and global efficiency, respectively. *Q*, *β*, and *r* denote modularity, hierarchy, and assortativity, respectively.

Figure 5 shows the TRT reliability of 10 global network metrics against the increasing sparsity thresholds, from 0.01 to 0.99, stepped by 0.01. In general, the reliability of the network metrics manifested considerable variation across the sparsity threshold values. The reliability also displayed moderate variation across different network metrics at the same thresholds, irrespective of the HbO, HbR, and HbT signals. The reliability of most of the network metrics was maintained at a fair to excellent level over a broad threshold range for the three hemoglobin signals. For example, global efficiency *E_glob_* exhibited fair to excellent reliability in the threshold ranges of 0.1 to 0.7 for each hemoglobin signal.

**Figure 6:**
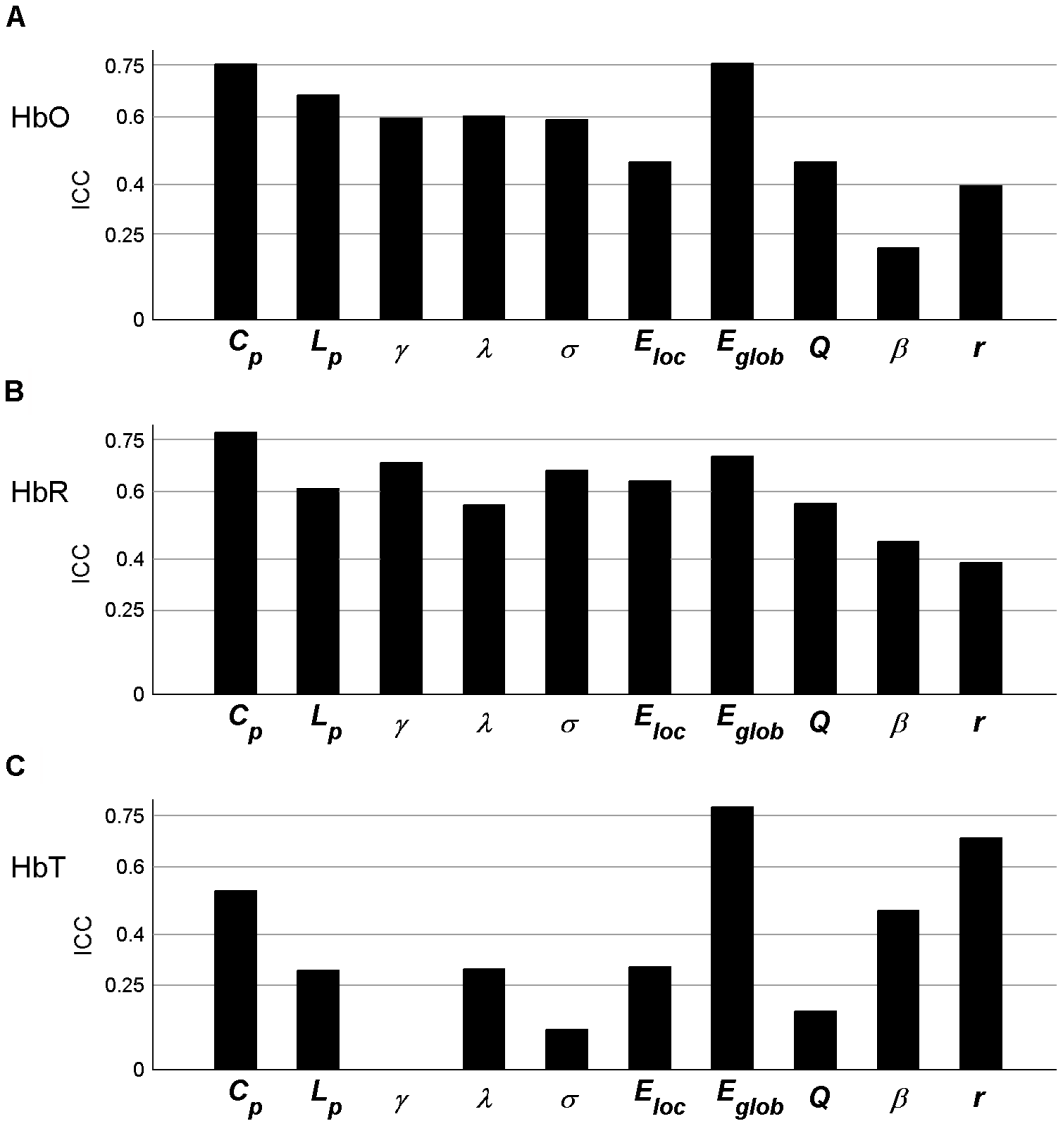
Threshold-independent reliability analysis of global network metrics. The areas under the curves (AUCs) of each global metric were used to provide threshold-independent reliability evaluation. (A–C) The global metric reliability was derived from HbO, HbR, and HbT, respectively. *C_p_*, *L_p_*, *γ*, *λ,* and σ denote the clustering coefficient, characteristic path length, normalized clustering coefficient, normalized characteristic path length, and small-world, respectively. *E_loc_* and *E_glob_* denote local efficiency and global efficiency, respectively. *Q*, *β*, and *r* denote modularity, hierarchy, and assortativity, respectively.

Additionally, the AUC was calculated to test for significant differences in each metric across subjects between sessions. The results indicated that almost all parameters (except *β* in HbR) were not significantly different (paired t-tests, *p*>0.05, Table 2), suggesting a high similarity across global network measures between sessions at the individual level. The AUC of each metric was also employed to calculate the reliability measure. The results of the reliability analyses are shown in Fig. 6A–C for HbO, HbR, and HbT, respectively. For both HbO and HbR signals, almost all network metrics consistently exhibited fair to excellent reliability (except *β* in HbO and *r* in HbR showing poor and low reliability, respectively). In contrast, four of ten metrics showed fair to excellent reliability for HbT (Fig. 6C). Numerically, the *ICC* values for HbR were the highest (*ICC* = 0.60, averaged across all metrics), followed by HbO (mean *ICC* = 0.55); HbT had the lowest relative *ICC* values (mean *ICC* = 0.36). Further statistical analysis using a one-way repeated measures ANOVA revealed that the reliability of all network metrics from the three different hemoglobin concentration signals exhibited significant differences [F(2, 9)  = 5.20; *p* = 0.017] and that HbR generally displayed the best relative reliability (paired t-tests, *p* = 0.03). Viewed from a single network metric, the clustering coefficient *C_p_* and global efficiency measure *E_glob_* displayed relatively better reliability, with an average reliability level of excellent (*ICC* > 0.7) across the HbO, HbR, and HbT signals.

**Table 2:** Significant differences in the global network metric (across subjects) between sessions revealed by a paired t-test.

Global	HbO		HbR		HbT	
network metrics	*t*	*p*	*t*	*p*	*t*	*p*
***C_p_***	−0.12	0.91	0.68	0.51	0.82	0.42
***L_p_***	−0.05	0.96	0.24	0.82	−1.13	0.27
***γ***	1.80	0.09	0.53	0.60	0.36	0.72
***λ***	−1.28	0.22	−0.84	0.42	0.08	0.94
***σ***	1.99	0.06	0.43	0.67	0.43	0.67
***E_loc_***	0.06	0.95	0.52	0.61	1.15	0.26
***E_glob_***	−0.15	0.88	−0.29	0.78	0.52	0.61
***Q***	0.65	0.53	−0.26	0.80	0.88	0.39
***β***	1.31	0.21	2.44	0.03	1.37	0.19
***r***	−0.25	0.81	−2.63	0.02	−0.07	0.94

The AUC of each global metric was used as the statistical variable.

### TRT reliability of nodal metrics

**Figure 7:**
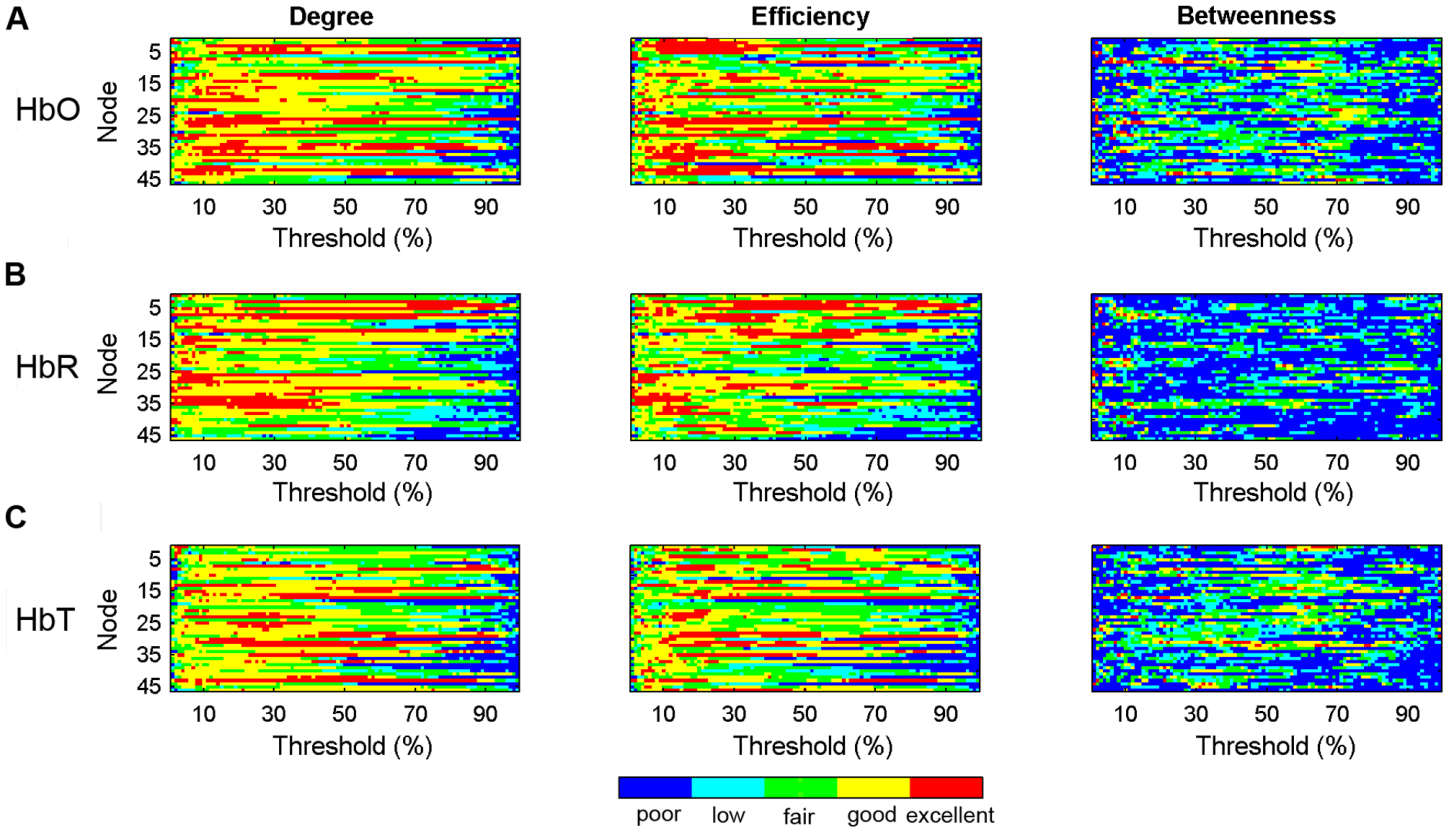
TRT reliability of nodal centrality metrics as a function of sparsity threshold. (A–C) The nodal metric reliability was derived from HbO, HbR, and HbT, respectively. The five colors correspond to five different reliability grades: red, yellow, green, cyan, and blue represent excellent (0.75< *ICC* <1), good (0.6< *ICC* <0.75), fair (0.4< *ICC* <0.6), low (0.25< *ICC* <0.4), and poor (*ICC*<0.25) reliability of the nodal centrality metrics, respectively.

**Figure 8:**
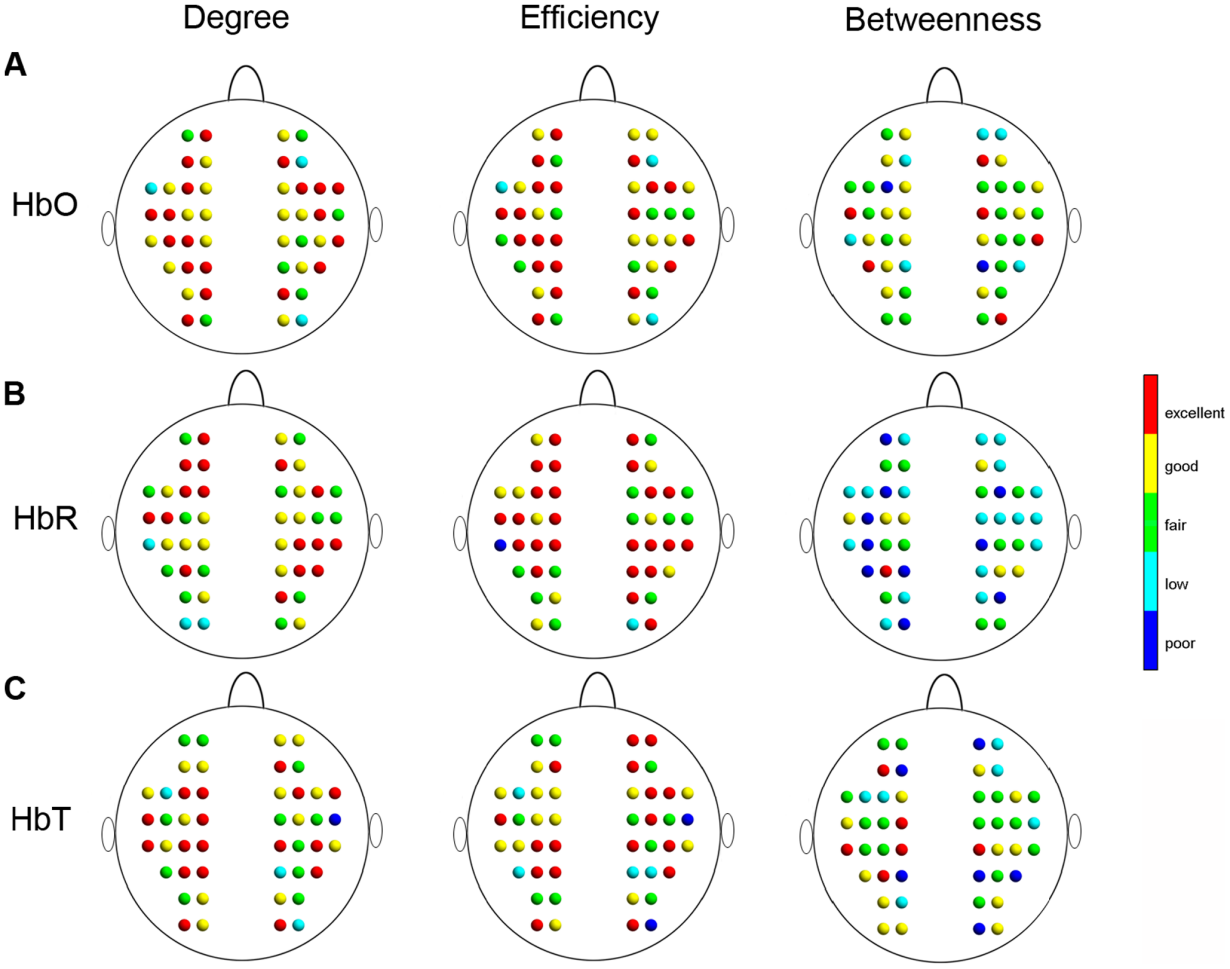
Threshold-independent reliability analysis of nodal centrality metrics. The areas under the curves (AUCs) of each nodal metric were used to provide threshold-independent reliability evaluation. (A–C) The nodal reliability was derived from HbO, HbR, and HbT, respectively. Different colors in the nodes correspond to different reliability grades: red, yellow, green, cyan, and blue colors represent excellent (0.75< *ICC* <1), good (0.6< *ICC* <0.75), fair (0.4< *ICC* <0.6), low (0.25< *ICC* <0.4), and poor (*ICC*<0.25) reliability of the nodal centrality metrics, respectively.

Figure 7 shows the nodal reliability of the degree, efficiency and betweenness measures as a function of the sparsity threshold values. Similar to global network metrics, the reliability of the nodal metrics also changes with sparsity and varies between metrics. Visually, betweenness exhibited the worst reliability across the range of thresholds, while the reliabilities of degree and efficiency were higher and comparable to one another for the HbO, HbR, and HbT signals. Furthermore, nodal reliability was estimated based on a threshold-independent metric, the AUC of each nodal metric. Figure 8 shows the reliability distribution of the total 46 nodes on one brain image. The nodes with fair to excellent reliability held ∼90% (41/46) of the total nodes and were distributed widely throughout the whole brain (Figs. 8A and 8B), suggesting that nodal reliability is not related to the spatial position of the nodes in brain regions.

**Figure 9:**
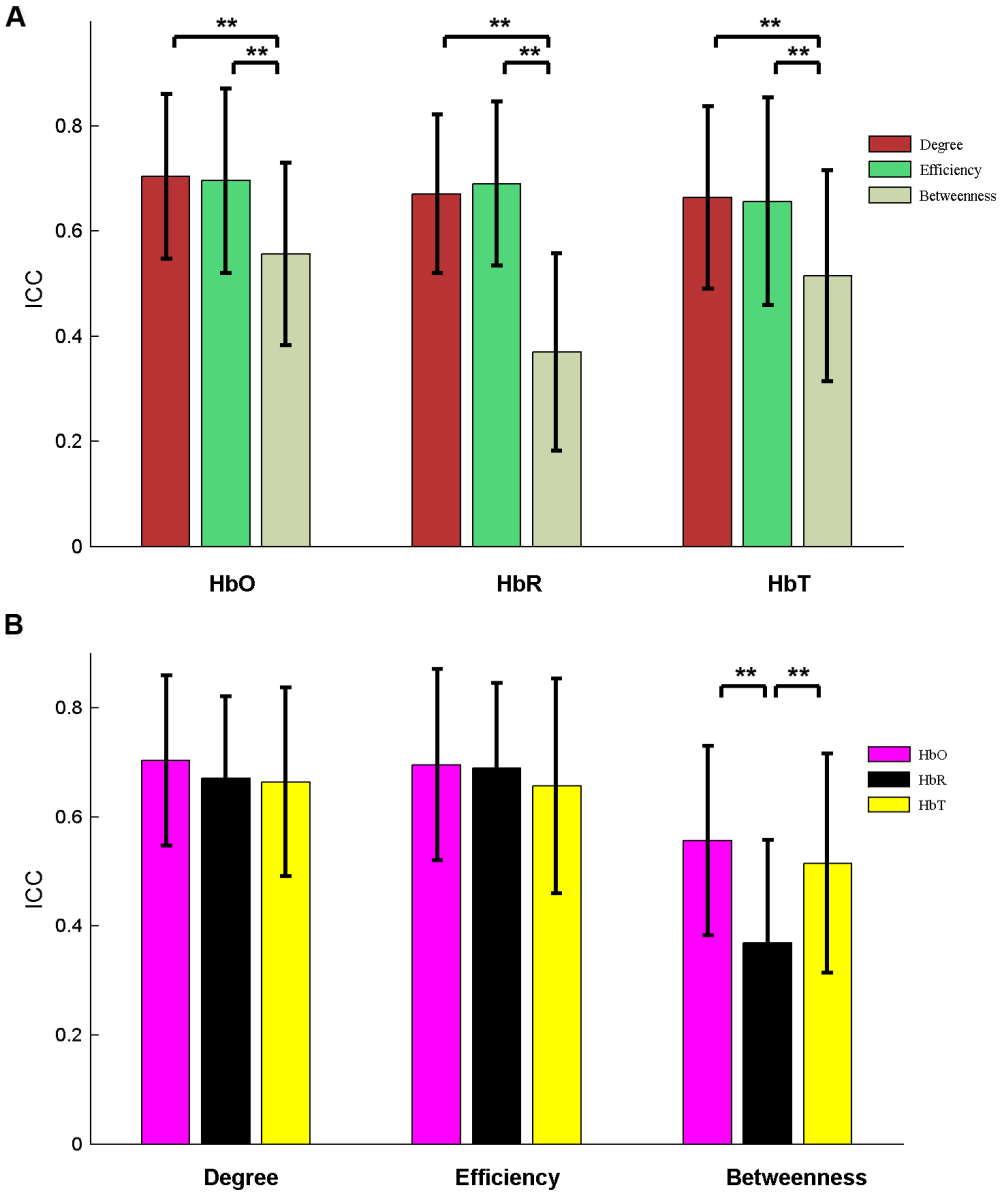
Significant differences (paired t-test) in nodal metric reliability. (A) Reliability for three nodal centrality metrics (degree, efficiency, and betweenness). (B) Reliability for three concentration signals (HbO, HbR, and HbT). Note that the reliability of nodal degree and efficiency is concordant across HbO, HbR, and HbT and is also significantly (*p*<0.0001) higher than that of nodal betweenness. Error bars correspond to the standard deviation of the mean across the total nodes. The double asterisk indicates *p*<0.0001.

We further performed a two-factor repeated measures ANOVA to investigate the effects of different nodal measures (degree, efficiency and betweenness) and different concentration signals (HbO, HbR and HbT) on nodal reliability. The AUC-based *ICC* values were used for this statistical analysis. The results showed a significant main effect of network measures [*F* (2, 45)  = 63.47, *p*<0.0001] (Fig. 9A) and a significant main effect of concentration signals [*F* (2, 45)  = 5.54, *p* = 0.0054] (Fig. 9B). There was also a significant interaction between network measures and concentration signals [*F* (2, 45)  = 11.79, *p*<0.0001], indicating that the reliability of nodal network measures was not consistent across all concentration signals. Post hoc comparisons (paired t-tests) showed that the reliability of degree and efficiency was concordant and was higher than that of betweenness (*p*<0.0001) for HbO, HbR, and HbT. The high reliability of degree and efficiency did not significantly differ among these three concentration signals (*p* > 0.05), suggesting the robustness of degree and efficiency for varying types of hemoglobin information.

### TRT reliability of network metrics after denoising using ICA

**Figure 10:**
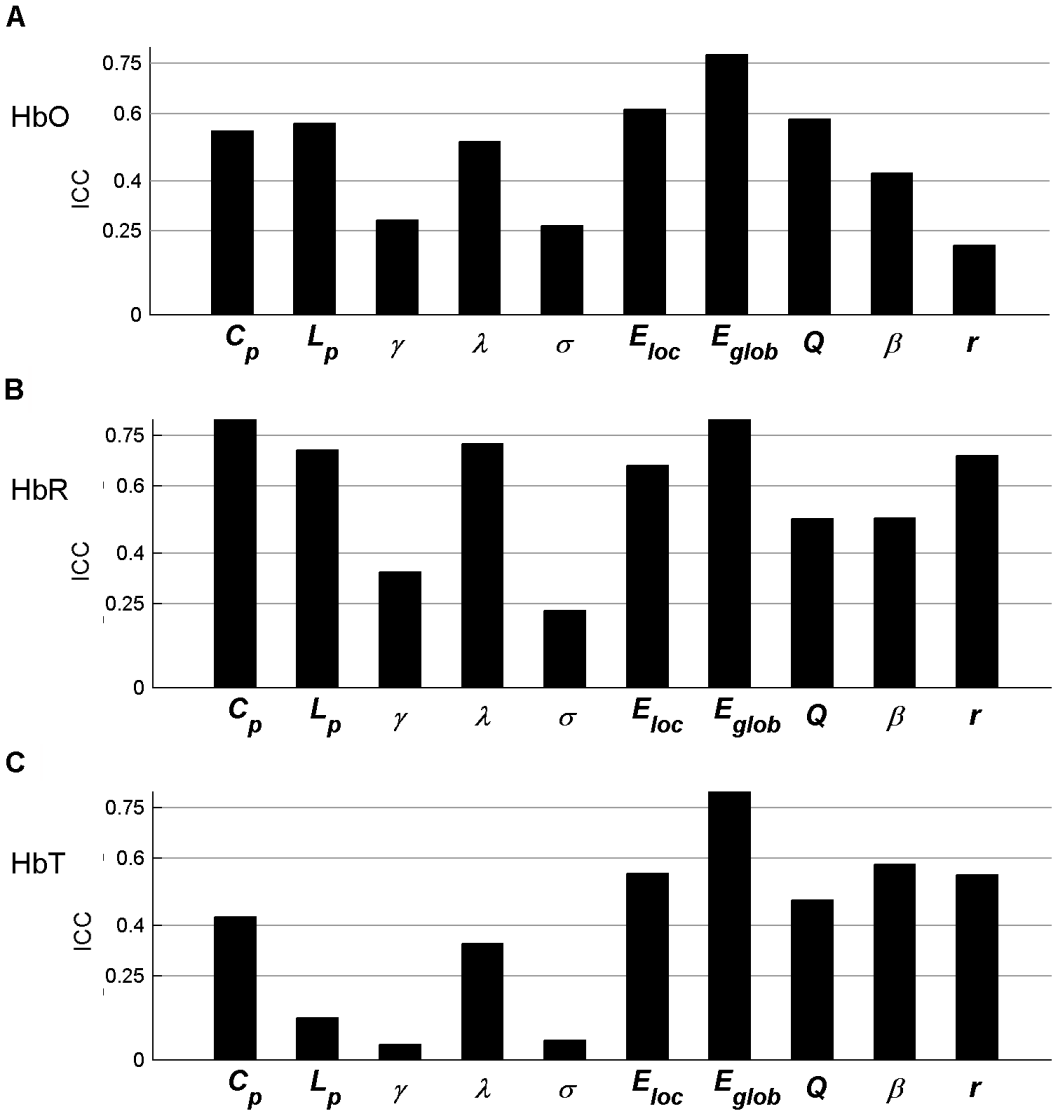
Threshold-independent reliability analysis of ICA- derived global network metrics. The areas under the curves (AUCs) of each global metric were used to provide threshold-independent reliability evaluation. (A–C) The global metric reliability was derived from HbO, HbR, and HbT, respectively. *C_p_*, *L_p_*, *γ*, *λ,* and σ denote the clustering coefficient, characteristic path length, normalized clustering coefficient, normalized characteristic path length, and small-world, respectively. *E_loc_* and *E_glob_* denote local efficiency and global efficiency, respectively. *Q*, *β*, and *r* denote modularity, hierarchy, and assortativity, respectively.

**Figure 11:**
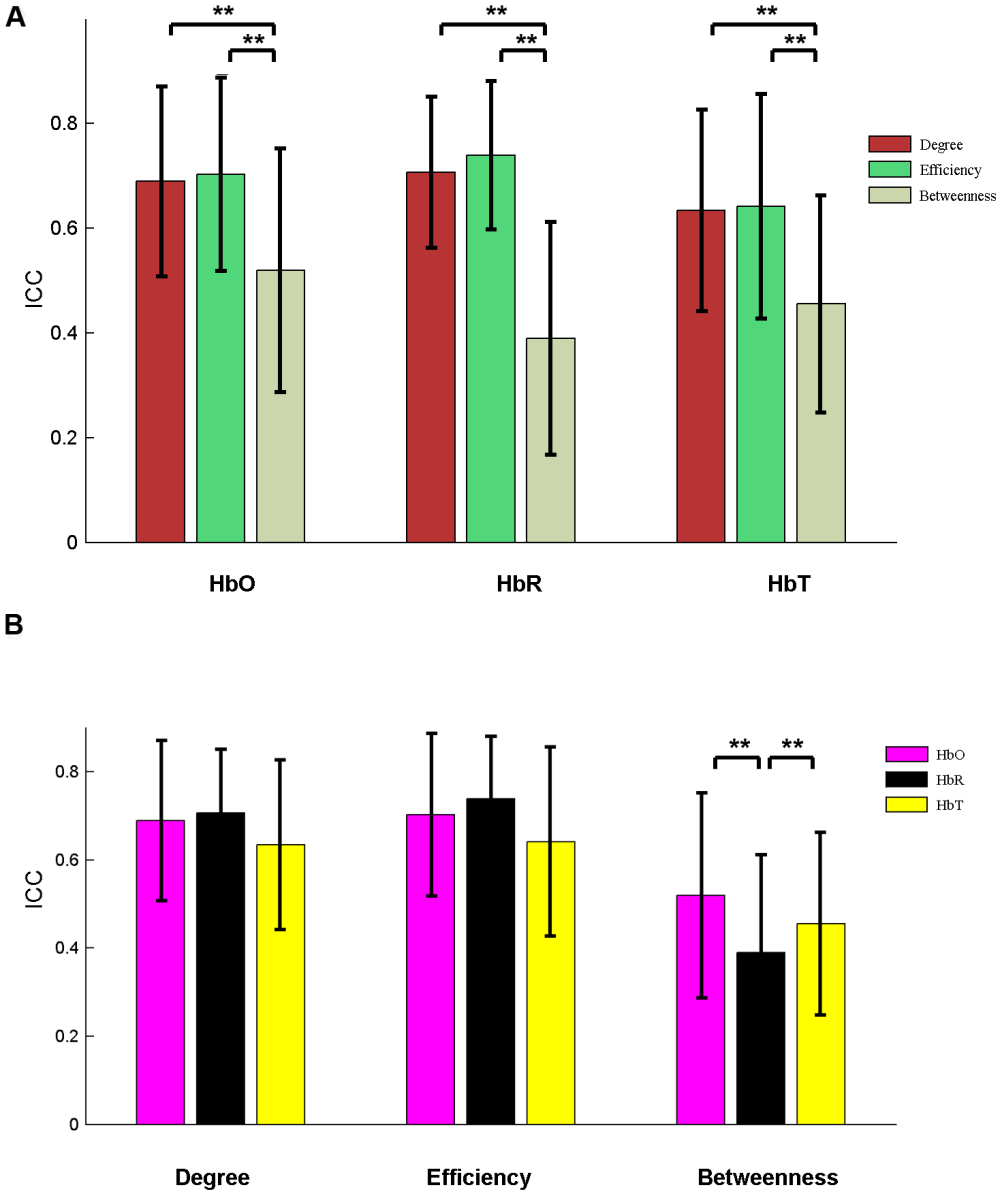
Significant differences (paired t-test) in ICA-derived nodal metric reliability. (A) Reliability for three nodal centrality metrics (degree, efficiency, and betweenness). (B) Reliability for three concentration signals (HbO, HbR, and HbT). Each of these three concentration signals was denoised using ICA. Note that the reliability of nodal degree and efficiency is concordant across HbO, HbR, and HbT and is also significantly (*p*<0.0001) higher than that of nodal betweenness. Error bars correspond to the standard deviation of the mean across the total nodes. The double asterisk indicates *p*<0.0001.

All the results of network analysis after denoising using ICA are presented in Figs. 10–11, Figs. S1, S2, S3, S4, S5 in Supporting Information S1, and Tables S1–S2 in Supporting Information S1. In general, we found that the main results remained few changes as compared to those without ICA analysis. For example, the *ICC* values of RSFC maps with ICA denosing were 0.63±0.20 for HbO, 0.68±0.20 for HbR, and 0.71±0.21 for HbT, which was highly compatible with the results without ICA analysis (HbO: 0.70±0.15; HbR: 0.65±0.19; HbT: 0.71±0.17) (Figs. S1–S2 in Supporting Information S1). Meanwhile, at the individual level, the RSFC maps still remain a high correlation between two sessions after denoising using ICA (Table S1 in Supporting Information S1). For global and nodal network metrics, the reliability was threshold-sensitive and varied among both network metrics and hemoglobin concentration signals (Figs. 10–11 and Figs. S3, S4, S5 in Supporting Information S1), which was highly consistent with the results without ICA denosing. Specifically, the *ICC* values for HbR were the highest (*ICC* = 0.60, averaged across all metrics), followed by HbO (mean *ICC* = 0.48) and HbT (mean *ICC* = 0.40). Moreover, the global efficiency *E_glob_* still displayed the highest reliability (the average *ICC* = 0.83 across the HbO, HbR, and HbT signals) among all 10 global metrics (Fig. 10 and Fig. S3 in Supporting Information S1) and both the nodal degree and efficiency centralities showed higher *ICC* values than the betweenness centrality (*p*<0.0001) for all three hemoglobin concentration signals (Fig. 11 and Figs. S4–S5 in Supporting Information S1). There were no significant differences in the ICA-derived global network metrics between sessions (Table S2 in Supporting Information S1). Notably, we also observed small variations on the reliability magnitude of several network metrics with ICA-based denoising. For example, the *IC*C values of *E_loc_* and *E_glob_* slightly increased for HbO, HbR, and HbT signals, whereas the ICC values of *C_p_* and *L_p_* slightly decreased for HbO and HbT signals, as compared to the results without ICA denosing (Fig. 6 vs. Fig. 10).

## Discussion

Previously, we showed that R-fNIRS can be used in combination with graph theory methodology to reveal topological properties of human functional brain networks [12]. In the current study, we further addressed the TRT reliability of these topological properties. A comprehensive assessment of network reliability was carried out on three sets of network measures (RSFC, global network metrics and regional nodal metrics). These network metrics were calculated based on two sets of noise-reduction data using frequency-based and ICA-based denoising approaches, respectively, and the results consistently demonstrated that the R-fNIRS brain networks possessed good reliability on RSFC and fair to excellent reliability on most global and nodal network metrics.

### TRT reliability of RSFC maps

Based on a correlational analysis and a reliability assessment, we demonstrated that the fNIRS-based RSFC maps have high similarity (Pearson correlation: *r* = 0.91±0.03, averaged across three concentration signals) and high reliability (*ICC* = 0.69±0.03, averaged across three concentration signals) across sessions. Our results are compatible with the findings of previous RSFC reliability studies based on resting-state fMRI [51], [52] and R-fNIRS [24] data. For example, Shehzad and colleagues [51] found that the fMRI-derived RSFC maps showed remarkable spatial consistency and modest to good reliability across different scanning sessions for both region-of-interest and voxel-wise analyses. The high reliability of RSFC maps were also further confirmed by Wang et al. [52] at a whole-brain level. Using R-fNIRS data, Zhang et al. [24] demonstrated that the RSFC of the sensorimotor regions was TRT reliable at both individual and group levels within and between sessions. In the present study we showed the high reliability of whole-brain RSFC maps based on the R-fNIRS data. Collectively, these accumulated evidences demonstrated that the measure of RSFC is reliable and trustworthy to measure the brain's intrinsic functional architecture regardless of the imaging techniques of fNIRS and fMRI. Additionally, we found positive correlation between RSFC reliability and connectivity strength for HbO and/or HbR. This finding suggests that the reliability of functional connectivity for these two concentration signals is partly determined by their connection strength.

### TRT reliability of global network metrics

In recent years, TRT reliability of global network metrics has been examined using different imaging modalities, including MEG and fMRI techniques [52], [53], [54], [55], [56]. These research findings provide solid foundation for potential applied studies on longitudinal changes, measurements of normal development, or drug treatment-related effects. In the present study, we investigated the TRT reliability of global metrics of R-fNIRS brain networks from two perspectives. First, we examined the effect of varying threshold values on network reliability and found that the reliability of all global metrics is related to those selected threshold values. The threshold-sensitive characteristics of global network metrics are considered as typical concerns in brain network study, and have also been demonstrated in previous fMRI studies [52], [53], [54]. For example, Wang and colleagues [52] pointed out that the reliability of global network metrics varied from poor to excellent grades which depended on the selected threshold values. These observations suggest that it is extremely important to select an appropriate threshold for obtaining reliable results in the study of brain networks. However, it needs to notice that there is currently no accepted standard in the literature for determining the suitable threshold values applied to different network studies. Generally, a compromise method is to investigate the network over a continuous threshold range from the sparsest connection to the densest connection.

**Figure 12:**
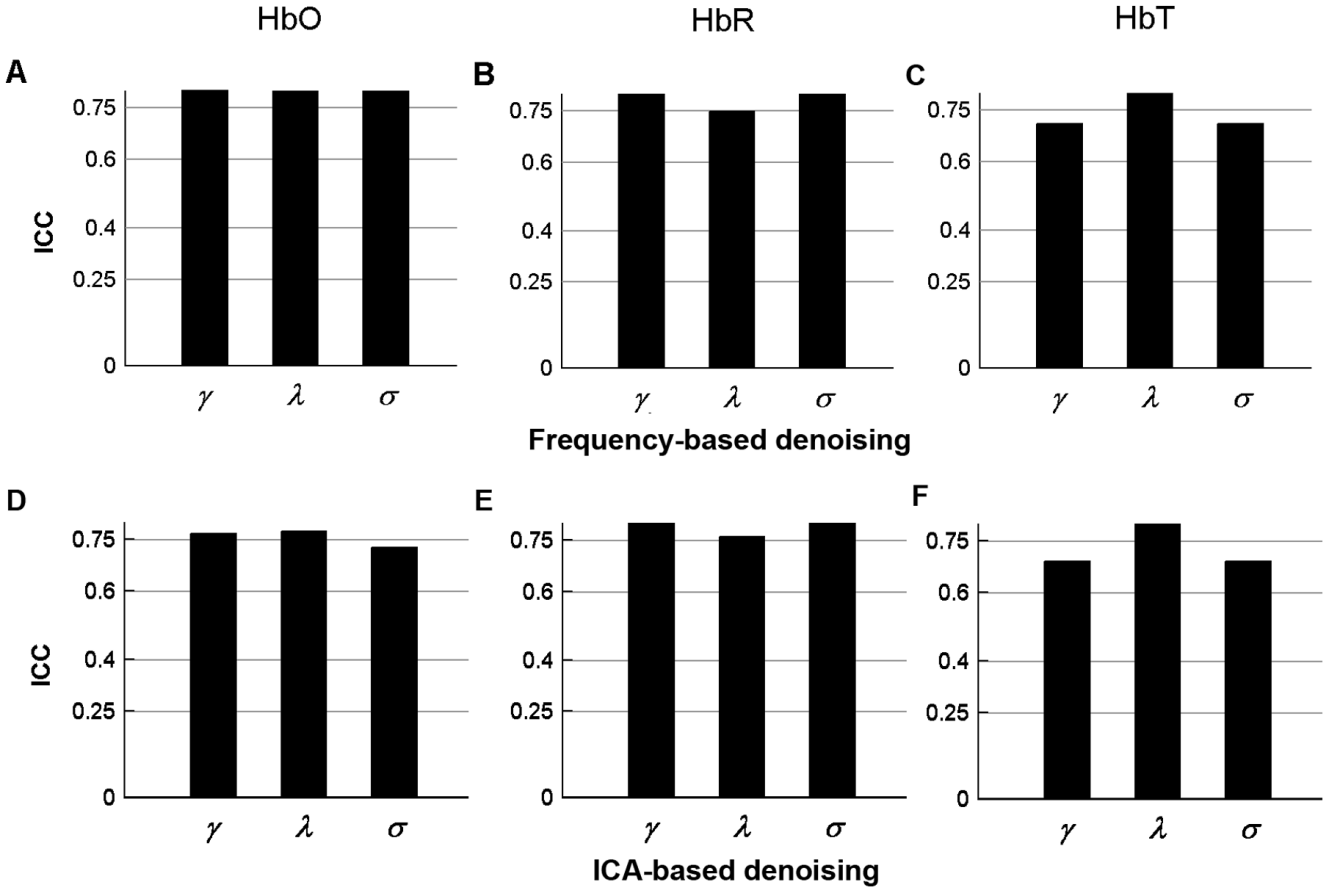
Reliability analysis of global network metrics derived from the correlation matrix randomization method. The areas under the curves (AUCs) of each global metric were used to provide threshold-independent reliability evaluation. (A–C) show the frequency-derived (i.e. band-pass filter) global metric reliability and (D–F) show the ICA-derived global metric reliability.*γ*, *λ,* and σ denote the normalized clustering coefficient, normalized characteristic path length, and small-world, respectively.

Second, we examined the TRT reliability of global network metrics by excluding the impact of thresholds and found that most global network metrics exhibited overall high TRT reliability. Of note, our observation is consistent with a previous report from Telesford et al., [56], who demonstrated the TRT reliability of graph metrics of functional human brain network based on a set of BOLD-fMRI data and also investigated an overall high TRT reliability on some global network metrics such as the clustering coefficient *C_p_*, path length *L_p_*, and global efficiency *E_glob_*. However, it is worth noting that these findings are inconsistent with those shown in two previous BOLD-fMRI studies demonstrating low [52] and modest [53] TRT reliability for the global network metrics, respectively. For example, Wang et al. [52] found that most of the global network metrics displayed overall low TRT reliability, especially for the clustering coefficient *C_p_* that uniformly showed poor reliability (*ICC*<0.25) regardless of which type of network (binarized or weighted) adopted for that study. By contrast, Braun et al. [53] found that the reliability of global network metrics was moderate (0.41< *ICC* <0.59) but highly depended on the processing strategies such as the selection of frequency-band range and the use of global signal regression. For the discrepancies among these studies, we speculate that it could be attributable to the different imaging modalities, scanning intervals, network size, population size, and the node and edge definitions applied in these studies. Moreover, the resting-state imaging data captures the information of complex integration among various brain regions, and the integration always exhibits a dynamic, time-varying fashion on the order of second or minute [57]. In the future, it would be interesting to compare TRT reliability of graphic metrics of brain functional networks derived from the technique of simultaneous acquisition of different modalities (e.g., fNIRS vs. BOLD-fMRI)**.** In addition, it is worth noting that there also exist several global network metrics exhibiting low reliability across different fNIRS signals (i.e., HbO, HbR, and HbT), such as the characteristic path length *Lp* (Fig.10C), hierarchy (Fig. 6A), assorsitivity (Fig. 10A), and modularity parameter *Q* (Fig.10C).The possible reasons for the low reliability global-metrics could be 1) that these network metrics depend on the fine structure of observed networks, while the fine structure could be sensitive to the subtle fluctuations of functional connections between two sessions; 2) that these network metrics characterizing aspects of intrinsic brain function that can be less robustly estimated using fNIRS signal(s) [53]; 3) that these network metrics manifested a low between-subject variance in differentiating subjects or a high within-subject variance across scanning sessions [52]. Moreover, we also found that the normalized metrics of normalized clustering coefficient *γ* (Figs. 6C and 10C), normalized characteristic path length *λ* (Figs. 6C and 10C) and small-world scale σ (Figs. 6C, 10B, and 10C) also exhibited low reliability across HbO, HbR and/or HbT signals. We speculated that the low reliability of these three network metrics might be related to the randomization approaches which were used to generate the matched random networks. To examine this point, we recalculated these three global metrics (normalized clustering coefficient, normalized characteristic path length, and small-worldness) using the correlation matrix randomization method that preserves the transitive network structures [58]. Likewise, the reliability level of these three metrics was also quantified by *ICC*. We found that the reliability of these three metrics was largely improved (Fig. 12) as compared to the results obtained from that topology randomization method (Fig. 6 and Fig. 10). The improvement of reliability could be attributable to decreased within-subject variance due to the conservative estimation of global metrics induced by the correlation matrix randomization algorithm. This observation also suggested that it would be important to choose appropriate randomization methods for achieving reliable results of brain network analysis.

### TRT reliability of nodal metrics

Similar to the findings in global network measures, we also observed that nodal reliability was threshold-sensitive for local nodal metrics. This finding further highlights the importance of threshold selection for achieving reliable results. Additionally, regarding to the reliability assessment of nodal centrality metrics, a previous fMRI study [52] has made a systematic investigation on 6 nodal metrics (including the nodal degree, efficiency, betweenness, cluster coefficient, participant coefficient, and normalized participant coefficient), and found that the nodal degree had the highest TRT reliability. In the current study, we found that in addition to the nodal degree, the nodal efficiency also exhibited fairly high reliability, and both were significantly higher than that reliability of betweenness regardless of the HbO, HbR or HbT signals. More importantly, the reliability of both nodal degree and nodal efficiency exhibited no significant differences across the different hemoglobin concentration signals, suggesting that both these metrics are robust to different hemodynamic contrasts. Similarly, in the previous BOLD-fMRI study [52], the authors also found that the reliability of nodal network properties was minimally affected by many factors such as scanning time interval, network membership (networks excluding or including negative correlations) and network type (binarized or weighted networks). These findings indicated that the reliability of nodal properties (e.g., nodal degree and efficiency) was more robust and least affected, as compared to that of global network metrics. However, we also noticed that among the nodal centrality metrics, the nodal betweenness was the most sensitive to spatial nodal configuration and most unreliable across different concentration contrasts, which indicated that the nodal betweenness metric should be used with caution if no additional effort was made to improve its reliability. This observation from fNIRS data was also proved to be consistent with the previous BOLD-fMRI results [52].

### Comparisons of graph metric reliability among the three hemoglobin concentrations

In the current study, we found that the three hemoglobin concentrations (HbO, HbR, and HbT) exhibited different reliability profiles for graph-based network metrics: the HbR showed the best global reliability (mean *ICC* = 0.604), while the HbT showed the worst reliability (*ICC*<0.4 for most global metrics). In our previous study [12], we also demonstrated that although all the three types of concentration signals can be used to characterize topological organization of intrinsic functional brain architecture, the quantitative network parameters differed significantly. The discrepancies among different concentration signals results may be attributable to their different nature in hemodynamic responses to transient neural activity and/or the difference in signal-to-noise ratio in the R-fNIRS measurements [23], [59]. While all of these three hemoglobin concentration signals are computed from the same raw R-fNIRS data, each signal reflects different hemodynamic response during neuronal activity. Under normal circumstances, the HbO signal reflects regional cerebral blood flow changes and the HbT signal reflects regional cerebral blood volume changes within the imaging field. HbR is generated through oxygen utilization in cerebral tissue. During fNIRS measurement, the signals recording the regional changes in cerebral blood flow and cerebral blood volume were generally aliased into various systemic physiological noises. The noise components were further propagated into different concentration signals during the calculation of hemoglobin concentration. It has been demonstrated that the HbR signal was generally minimally aliased into various noise components compared to the signals of HbO and HbT [60], [61], which, therefore, could bring about higher reliability for HbR signal. By contrast, both the HbO and HbT signals were more affected by changes in physiological conditions such as heart rate, blood pressure, blood flow, and breathing cycle [48], [60], [62], [63], which, to some extent, could reduce the reliability of the resultant network metrics.

In spite of the overall low reliability of global network metrics for HbT signal, we also noticed that several global metrics (e.g., hierarchy *β* and assortativity *r*) displayed fairly good reliability whereas they showed relatively lower reliability for the HbO and/or HbR signal (Fig. 6 and Fig. 10). This implies that the TRT reliability of some network metrics is specific to a certain concentration signal. Consequently, these findings highlight that fNIRS researchers should choose the optimum hemoglobin concentration contrast with caution for achieving reliable network investigation. Beyond the specificity of the high reliability of network properties on hemoglobin concentration, we also found that several network parameters (e.g., clustering coefficient *C_p_* and global efficiency measure *E_glob_*) exhibited excellent reliability on average across the three concentration signals. This suggests that *C_p_* and *E_glob_* could be utilized as two reliable biomarkers of functional brain architecture. In addition to these global metrics, we also found two nodal metrics: nodal degree and efficiency, which had mean *ICC* values across three concentration signals larger than 0.65, displayed not only the best reliability among all network measures but also the most robust performance among all network measures but also the most robust performance among the different hemoglobin concentration signals. These reliable and robust features suggest these metrics as reliable candidates to reveal the topological organization of intrinsic functional networks in future fNIRS studies in the human brain.

### Noise reduction in R-fNIRS data analysis

A common problem in R-fNIRS studies is that the spontaneous low-frequency fluctuations are contaminated by various different types of noise and artifacts such as instrumental noise, motion-induced artifacts, and physiological interferences which often occurs in the superficial layers of the head and in the brain tissue [62], [64], [65], [66]. These noises could have different sources and possess various frequency distributions of spectra. A general way to reduce the effect of these kinds of noise is the utilization of a band-pass filter with cut-off frequency from 0.009 to 0.08 Hz that represents the frequency range of hemodynamic signals thought to emanate from spontaneous neural activity [23], [27]. Using such a noise-reduction strategy, several R-fNIRS studies have explored intrinsic RSFC patterns in multiple brain networks involved in the sensorimotor [20], [23], [25], auditory [20], visual [23], [25], and language systems [67] as well as the whole-brain functional network [19]. The results from these studies are proved to be compatible with each other [20], [23], [25] and with previous fMRI investigations [27], [68], [69]. It suggests that the low-frequency spontaneous fluctuations measured in R-fNIRS signals may reflect spontaneous brain activity in spite of various confounding origins (e.g., systematic interferences in the superficial layers from skin and the scalp of the head). Nonetheless, the superficial interferences have been considered an important noise source in R-fNIRS data [70]. Recently, several denoising methods have been proposed for separating the noise from neuronal signals, which includes short separation measurement [71], [72], independent component analysis [48], and adaptive filter method [73], [74]. These methods are playing crucial roles in reducing the noise effects on the signals of spontaneous neural activity. Among them, both the short-distance measurement and the adaptive filter methods have a common experimental consideration that they need a design of additional short channels to record the superficial signal. However, a recent study has pointed out that the location of short separation measurement strongly impacts the performance of noise reduction in fNIRS [75]. Thus, it would be important and necessary to consider the number and the location of short-separation channels while adopting these two methods. In contrast, the ICA is a pure data-driven approach and does not require specific considerations of experimental design. Due to the capability of blind source separation, ICA can separate various noise and artifacts from measurement data, which have been widely used in both task-based and resting-state fMRI studies. In this study, we used the ICA approach for denoising and found that the reliability of many network metrics was not improved after the denoising processing. One reason could be attributable to the intrinsic instability of these network metrics [53], as estimated using fNIRS signal(s). Meantime, the characteristic of blind source separation of ICA makes the separated source components lack of explicit physiological explanation, which could lead to the difficulties of completely identifying and removing the noise components from the measured signals. Therefore, future studies are needed to test whether TRT reliability of brain functional networks could be improved by utilizing recording respiration and heart rates while collecting R-fNIRS data or other noise-reduction approaches (e.g., short-separation measurement).

### Further considerations

Several issues and limitations of the present study should be further addressed. First, the present study focused mainly on the evaluation of short-term reliability of R-fNIRS brain network metrics. Of note, one previous BOLD-fMRI study [52] found that the scanning interval had an influence on the network reliability and that long-term scans provided a higher TRT reliability than short-term scans on some network metrics such as normalized characteristic path length *λ*. Therefore, it would be our interests to examine the long-term TRT reliability in future fNIRS brain network studies. Second, several potential factors may affect the assessment of reliability of R-fNIRS brain networks but have not been elucidated in the present study, which includes: variation in fNIRS measurement environment (e.g., natural light and dark conditions) [76], variation in probe configuration across different imaging systems [77], variation in probe placement across participants and across scanning sessions[78], and potential change in participants' mental states during resting-state scanning [51]. In the future, the effects of these factors need to be systematically and comprehensively investigated. Third, we limited our examination of TRT reliability to widely used network metrics (10 global and 3 nodal characteristics). However, there are other network metrics, such as synchronization and vulnerability, whose TRT reliability should be evaluated in the future. Finally, there are different preprocessing strategies in fNIRS data analysis. Previous BOLD-fMRI studies have demonstrated that several factors (e.g., global signal removal, frequency bands and correlation metrics) might affect the network reliability [52], [55]. Future studies will be required to investigate the influence of different preprocessing techniques on network reliability within the context of R-fNIRS.

## Conclusions

We comprehensively evaluated the TRT reliability of graph metrics of R-fNIRS brain networks. Our results demonstrate that the R-fNIRS brain networks show good TRT reliability on RSFC and fair to excellent reliability on most global metrics and two nodal network metrics (nodal degree and efficiency). These findings suggest that the topological metrics with high reliability in R-fNIRS brain networks are potential to be employed in future studies of developmental and clinical neuroscience. Future research should also focus on the investigation and improvement of those network metrics that display low reliability.

## Supporting Information

Supporting Information S1 Figure S1, Spatial similarity of ICA-derived RSFC maps. Group-level RSFC maps for session 1 and session 2 and their Pearson correlation are displayed in the first to third columns. Figures (A) to (C) correspond to the RSFC data derived from HbO, HbR, and HbT, respectively. High similarity between sessions was observed in both the qualitative visual inspection and quantitative correlational analysis. Figure S2, Reliability analysis of ICA-derived RSFC maps. The first to third columns correspond to the data derived from HbO, HbR, and HbT, respectively. (A, B) The TRT reliability of RSFC maps and their corresponding reliability distributions. The reliability displays approximately normal configuration for all 1035 (i.e., 46×45/2) connections. The connections exhibit good reliability across HbO (mean ICC values 0.63), HbR (0.68) and HbT (0.65). (C) The relationship between RSFC strength and reliability as assessed by scatterplots. Each dot represents the group-level RSFC strength and the corresponding *ICC* value at the same connections. The trend lines were obtained by a linear least-squares fit method. Significant (*p*<0.05) *p*ositive correlations were found for HbO signals, suggesting stronger RSFC leads to higher reliability for this signal. Figure S3, TRT reliability of ICA-derived global network metrics as a function of sparsity threshold. (A–C) The global metric reliability was derived from HbO, HbR, and HbT, respectively. Five colors correspond to five different reliability grades. The red, yellow, green, cyan, and blue colors represent excellent ( 0.75< *ICC* <1), good (0.6< *ICC* <0.75), fair (0.4< *ICC* <0.6), low (0.25< *ICC* <0.4), and poor (*ICC*<0.25) reliability of global network metrics, respectively. *C_p_*, *L_p_*, *γ*, *λ,* and σ denote the clustering coefficient, characteristic path length, normalized clustering coefficient, normalized characteristic path length, and small-world, respectively. *E_loc_* and *E_glob_* denote local efficiency and global efficiency, respectively. *Q*, *β*, and *r* denote modularity, hierarchy, and assortativity, respectively. Figure S4, TRT reliability of ICA-derived nodal centrality metrics as a function of sparsity threshold. (A–C) The nodal metric reliability was derived from HbO, HbR, and HbT, respectively. The five colors correspond to five different reliability grades: red, yellow, green, cyan, and blue represent excellent (0.75< *ICC* <1), good (0.6< *ICC* <0.75), fair (0.4< *ICC* <0.6), low (0.25< *ICC* <0.4), and poor (*ICC*<0.25) reliability of the nodal centrality metrics, respectively. Figure S5, Threshold-independent reliability analysis of ICA-derived nodal centrality metrics. The areas under the curves (AUCs) of each nodal metric were used to provide threshold-independent reliability evaluation. (A–C) The nodal reliability was derived from HbO, HbR, and HbT, respectively. Different colors in the nodes correspond to different reliability grades: red, yellow, green, cyan, and blue colors represent excellent ( 0.75< *ICC* <1), good (0.6< *ICC* <0.75), fair (0.4< *ICC* <0.6), low (0.25< *ICC* <0.4), and poor (*ICC*<0.25) reliability of the nodal centrality metrics, respectively. Table S1, Pearson correlations at individual-level ICA-derived RSFC maps between sessions. Table S2, Statistical comparisons of the ICA-derived global network metrics (across subjects) between sessions.
(DOC)
