# Supplementary material for: Test-Retest Reliability of Graph Metrics in Functional Brain Networks: A Resting-State fNIRS Study
Source: PLoS One. 2013 Sep 9;8(9):e72425. doi: 10.1371/journal.pone.0072425 (PMC3767699; doi:10.1371/journal.pone.0072425)
Supplement: Supporting Information S1 — Figure S1, Spatial similarity of ICA-derived RSFC maps. Group-level RSFC maps for session 1 and session 2 and their Pearson correlation are displayed in the first to third columns. Figures (A) to (C) correspond to the RSFC data derived from HbO, HbR, and HbT, respectively. High similarity between sessions was observed in both the qualitative visual inspection and quantitative correlational analysis. Figure S2, Reliability analysis of ICA-derived RSFC maps. The first to third columns correspond to the data derived from HbO, HbR, and HbT, respectively. (A, B) The TRT reliability of RSFC maps and their corresponding reliability distributions. The reliability displays approximately normal configuration for all 1035 (i.e., 46×45/2) connections. The connections exhibit good reliability across HbO (mean ICC values 0.63), HbR (0.68) and HbT (0.65). (C) The relationship between RSFC strength and reliability as assessed by scatterplots. Each dot represents the group-level RSFC strength and the corresponding ICC value at the same connections. The trend lines were obtained by a linear least-squares fit method. Significant (p<0.05) positive correlations were found for HbO signals, suggesting stronger RSFC leads to higher reliability for this signal. Figure S3, TRT reliability of ICA-derived global network metrics as a function of sparsity threshold. (A–C) The global metric reliability was derived from HbO, HbR, and HbT, respectively. Five colors correspond to five different reliability grades. The red, yellow, green, cyan, and blue colors represent excellent ( 0.75< ICC <1), good (0.6< ICC <0.75), fair (0.4< ICC <0.6), low (0.25< ICC <0.4), and poor (ICC<0.25) reliability of global network metrics, respectively. Cp, Lp, γ, λ, and σ denote the clustering coefficient, characteristic path length, normalized clustering coefficient, normalized characteristic path length, and small-world, respectively. Eloc and Eglob denote local efficiency and global efficiency, respectively. Q, β, a [file pone.0072425.s001.doc]

**Supplementary materials**

**Table S1.** Pearson correlations at individual-level ICA-derived RSFC maps between sessions.

| **Subject** | **HbO** | |  | **HbR** | |  | **HbT** | |
| --- | --- | --- | --- | --- | --- | --- | --- | --- |
| **ID** | ***r*** | ***p*** |  | ***r*** | ***p*** |  | ***r*** | ***p*** |
| **1** | 0.55 | 1.10×10^-82^ |  | 0.48 | 1.87×10^-60^ |  | 0.76 | 7.67×10^-194^ |
| **2** | 0.63 | 6.40×10^-116^ |  | 0.65 | 4.38×10^-127^ |  | 0.63 | 1.84×10^-114^ |
| **3** | 0.49 | 1.89×10^-62^ |  | 0.53 | 4.55×10^-77^ |  | 0.47 | 4.20×10^-57^ |
| **4** | 0.75 | 3.27×10^-191^ |  | 0.55 | 3.75×10^-84^ |  | 0.68 | 3.05×10^-143^ |
| **5** | 0.74 | 6.85×10^-180^ |  | 0.75 | 4.65×10^-191^ |  | 0.60 | 3.20×10^-104^ |
| **6** | 0.61 | 4.72×10^-106^ |  | 0.73 | 8.22×10^-176^ |  | 0.62 | 5.84×10^-111^ |
| **7** | 0.59 | 1.20×10^-96^ |  | 0.77 | 5.16×10^-200^ |  | 0.74 | 8.55×10^-183^ |
| **8** | 0.72 | 3.07×10^-168^ |  | 0.62 | 3.14×10^-110^ |  | 0.56 | 1.41×10^-86^ |
| **9** | 0.84 | 2.12×10^-275^ |  | 0.92 | 0.00 |  | 0.69 | 8.92×10^-145^ |
| **10** | 0.82 | 2.15×10^-256^ |  | 0.52 | 1.13×10^-71^ |  | 0.90 | 0.00 |
| **11** | 0.62 | 2.00×10^-111^ |  | 0.60 | 3.09×10^-104^ |  | 0.73 | 3.02×10^-170^ |
| **12** | 0.65 | 9.02×10^-127^ |  | 0.65 | 8.79×10^-126^ |  | 0.54 | 3.90×10^-78^ |
| **13** | 0.67 | 4.86×10^-135^ |  | 0.54 | 3.03×10^-78^ |  | 0.46 | 2.94×10^-54^ |
| **14** | 0.77 | 1.95×10^-207^ |  | 0.71 | 1.85×10^-159^ |  | 0.74 | 5.21×10^-180^ |
| **15** | 0.54 | 1.00×10^-80^ |  | 0.57 | 2.99×10^-91^ |  | 0.54 | 6.93×10^-80^ |
| **16** | 0.70 | 2.36×10^-151^ |  | 0.79 | 1.41×10^-221^ |  | 0.73 | 1.54×10^-175^ |
| **17** | 0.84 | 1.80×10^-279^ |  | 0.86 | 2.52×10^-307^ |  | 0.78 | 8.07×10^-213^ |
| **18** | 0.36 | 1.57×10^-32^ |  | 0.62 | 5.48×10^-109^ |  | 0.43 | 1.47×10^-47^ |
| **mean** | **0.66** |  |  | **0.66** |  |  | **0.64** |  |
| **std** | **0.13** |  |  | **0.12** |  |  | **0.23** |  |

**Table S2.** Statistical comparisons of the ICA-derived global network metrics (across subjects) between sessions.

| **Global** | **HbO** | |  |  | **HbR** | |  |  | **HbT** | |  |
| --- | --- | --- | --- | --- | --- | --- | --- | --- | --- | --- | --- |
| **network metrics** | ***t*** | ***p*** |  |  | ***t*** | ***p*** |  |  | ***t*** | ***p*** |  |
| ***Cp*** | 0.08 | 0.94 |  |  | -0.17 | 0.87 |  |  | 0.08 | 0.94 |  |
| ***L_p_*** | -1.14 | 0.27 |  |  | -0.50 | 0.62 |  |  | -1.14 | 0.27 |  |
| ***γ*** | -0.27 | 0.79 |  |  | -0.45 | 0.66 |  |  | -0.27 | 0.79 |  |
| ***λ*** | -0.12 | 0.90 |  |  | -0.28 | 0.78 |  |  | -0.12 | 0.90 |  |
| ***σ*** | -0.29 | 0.77 |  |  | -0.37 | 0.72 |  |  | -0.29 | 0.77 |  |
| ***E_loc_*** | 0.76 | 0.46 |  |  | -0.64 | 0.53 |  |  | 0.76 | 0.46 |  |
| ***E_glob_*** | 1.16 | 0.26 |  |  | -1.27 | 0.22 |  |  | 1.16 | 0.26 |  |
| ***Q*** | 1.56 | 0.14 |  |  | -0.86 | 0.40 |  |  | 1.56 | 0.14 |  |
| ***β*** | 1.26 | 0.23 |  |  | -0.11 | 0.92 |  |  | 1.26 | 0.23 |  |
| ***r*** | -0.43 | 0.68 |  |  | 0.11 | 0.92 |  |  | -0.43 | 0.68 |  |

Paired t tests were performed on the AUC of each global metric to reveal between-session differences.


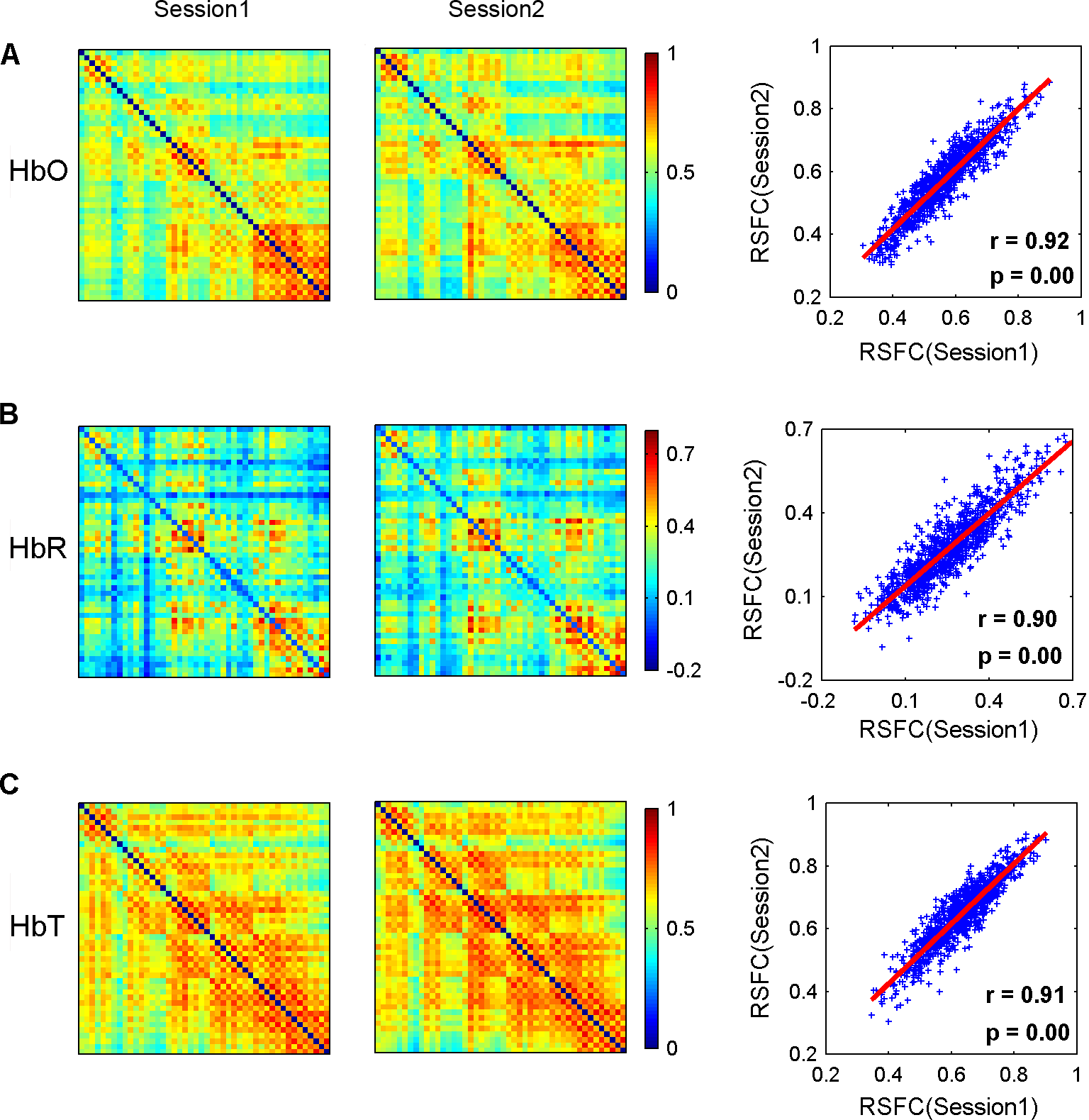


**Figure S1.** Spatial similarity of ICA-derived RSFC maps. Group-level RSFC maps for session 1 and session 2 and their Pearson correlation are displayed in the first to third columns. Figures (A) to (C) correspond to the RSFC data derived from HbO, HbR, and HbT, respectively. High similarity between sessions was observed in both the qualitative visual inspection and quantitative correlational analysis.


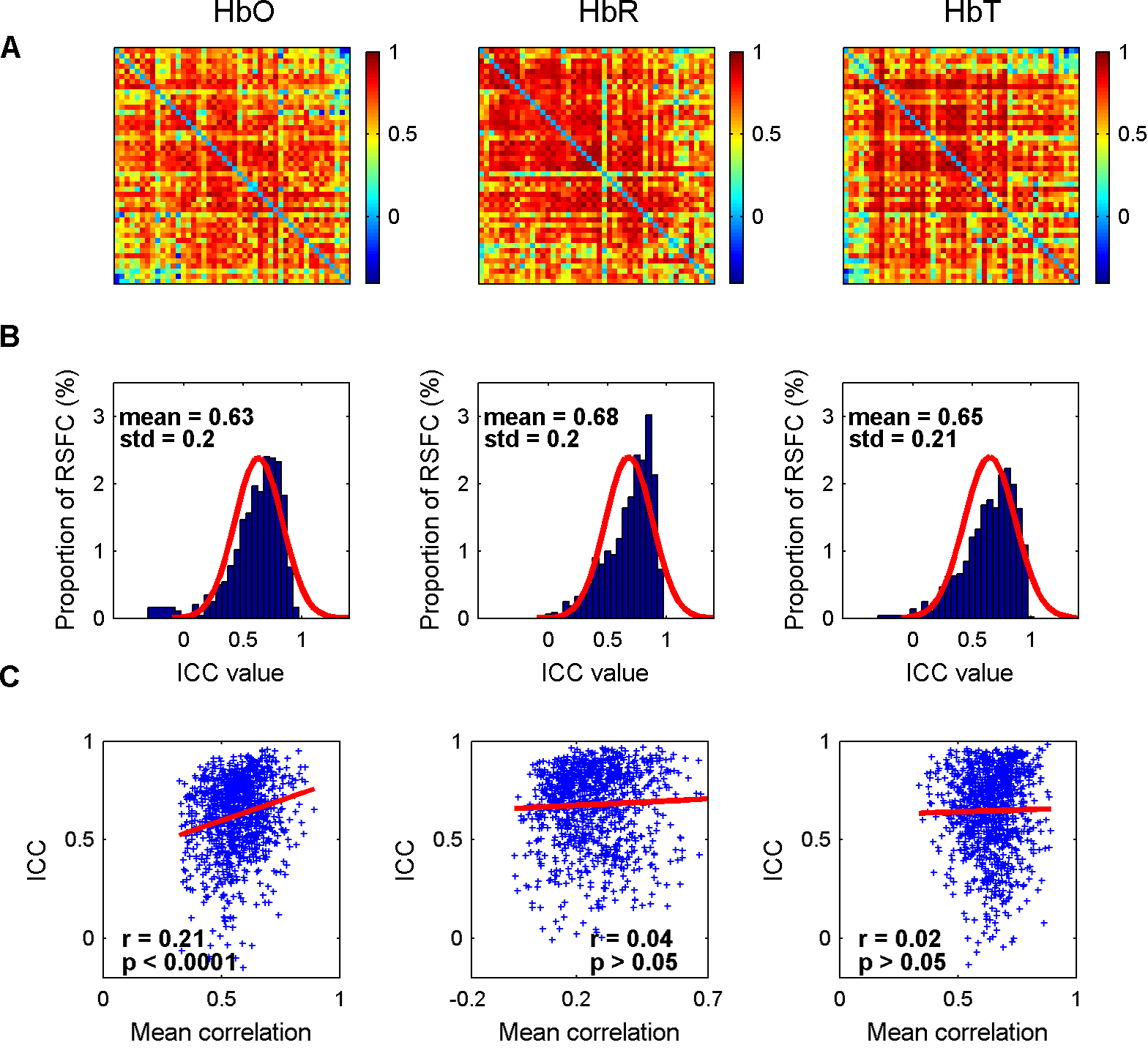


**Figure S2.** Reliability analysis of ICA-derived RSFC maps. The first to third columns correspond to the data derived from HbO, HbR, and HbT, respectively. (A, B) The TRT reliability of RSFC maps and their corresponding reliability distributions. The reliability displays approximately normal configuration for all 1035 (i.e., 46×45/2) connections. The connections exhibit good reliability across HbO (mean ICC values 0.63), HbR (0.68) and HbT (0.65). (C) The relationship between RSFC strength and reliability as assessed by scatterplots. Each dot represents the group-level RSFC strength and the corresponding *ICC* value at the same connections. The trend lines were obtained by a linear least-squares fit method. Significant (*P* < 0.05) positive correlations were found for HbO signals, suggesting stronger RSFC leads to higher reliability for this signal.


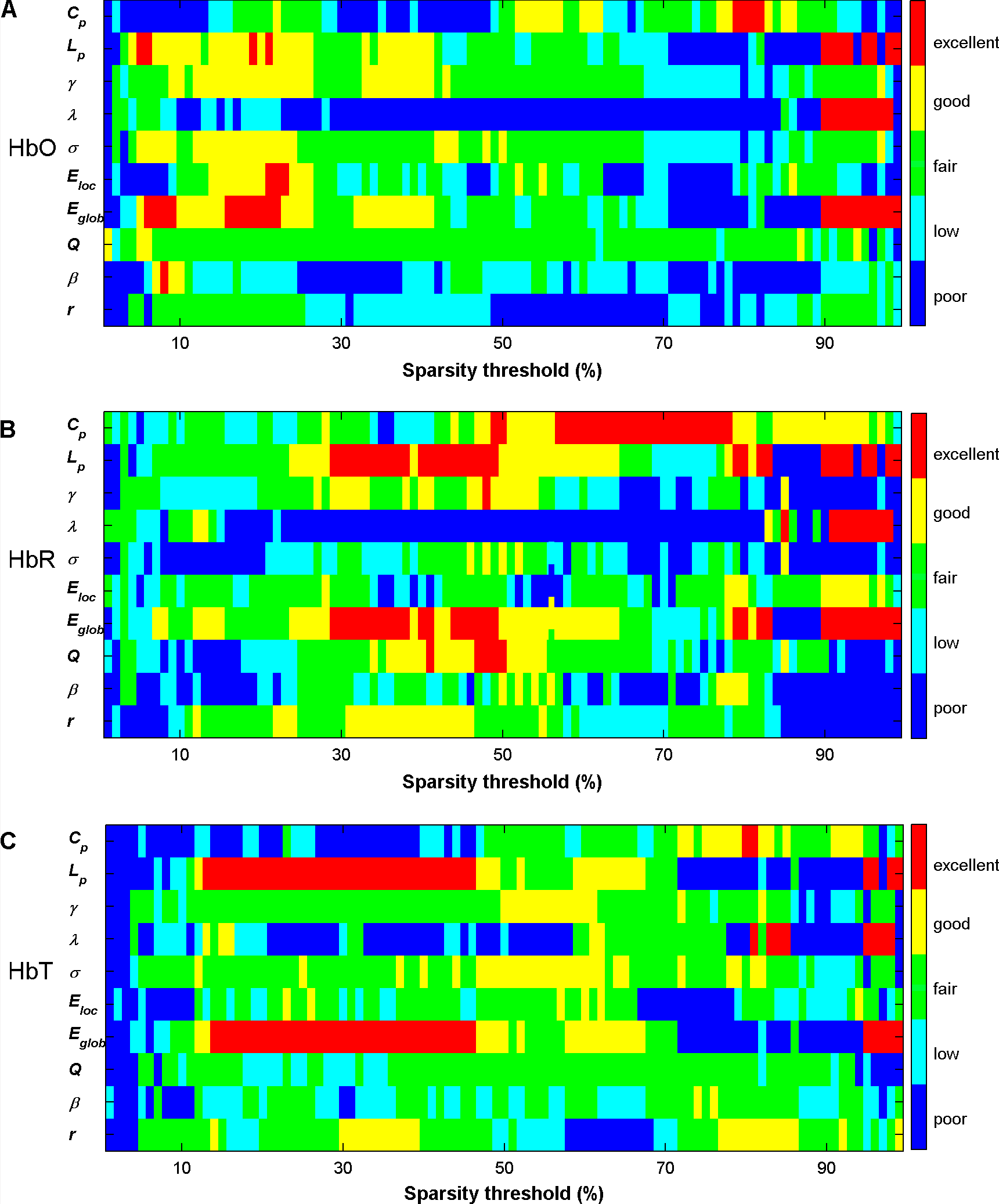


**Figure S3.** TRT reliability of ICA-derived global network metrics as a function of sparsity threshold. (A-C) The global metric reliability was derived from HbO, HbR, and HbT, respectively. Five colors correspond to five different reliability grades. The red, yellow, green, cyan, and blue colors represent excellent ( 0.75< *ICC* <1), good (0.6 < *ICC* < 0.75), fair (0.4 < *ICC* < 0.6), low (0.25 < *ICC* < 0.4), and poor (*ICC* < 0.25) reliability of global network metrics, respectively. *C_p_*, *L_p_*, *γ*, *λ,* and σ denote the clustering coefficient, characteristic path length, normalized clustering coefficient, normalized characteristic path length*,* and small-world, respectively. *E_loc_* and *E_glob_* denote local efficiency and global efficiency, respectively. *Q*, *β*, and *r* denote modularity, hierarchy, and assortativity, respectively.


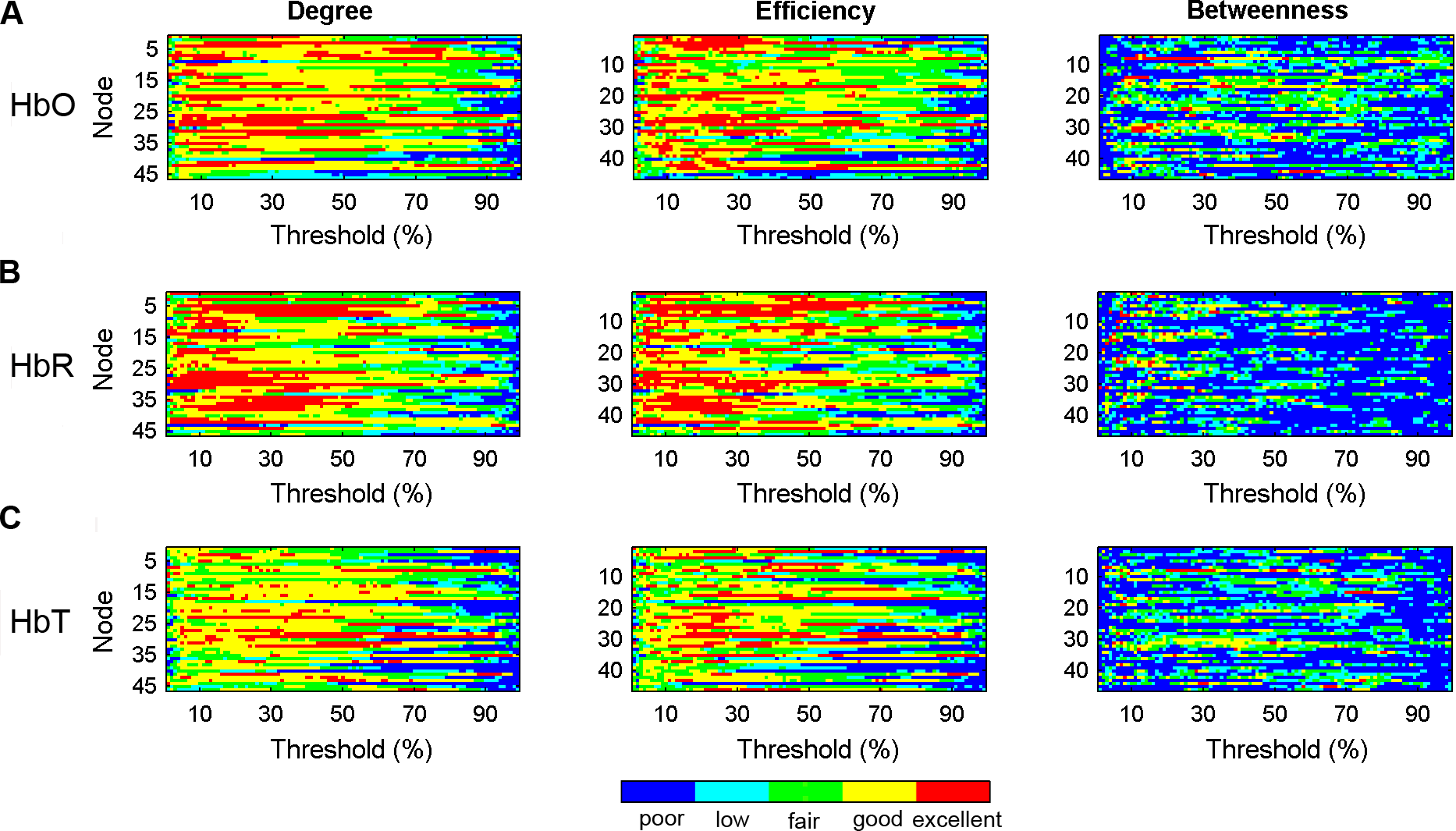


**Figure S4.** TRT reliability of ICA-derived nodal centrality metrics as a function of sparsity threshold. (A-C) The nodal metric reliability was derived from HbO, HbR, and HbT, respectively. The five colors correspond to five different reliability grades: red, yellow, green, cyan, and blue represent excellent (0.75< *ICC* <1), good (0.6 < *ICC* < 0.75), fair (0.4 < *ICC* < 0.6), low (0.25 < *ICC* < 0.4), and poor (*ICC* < 0.25) reliability of the nodal centrality metrics, respectively.


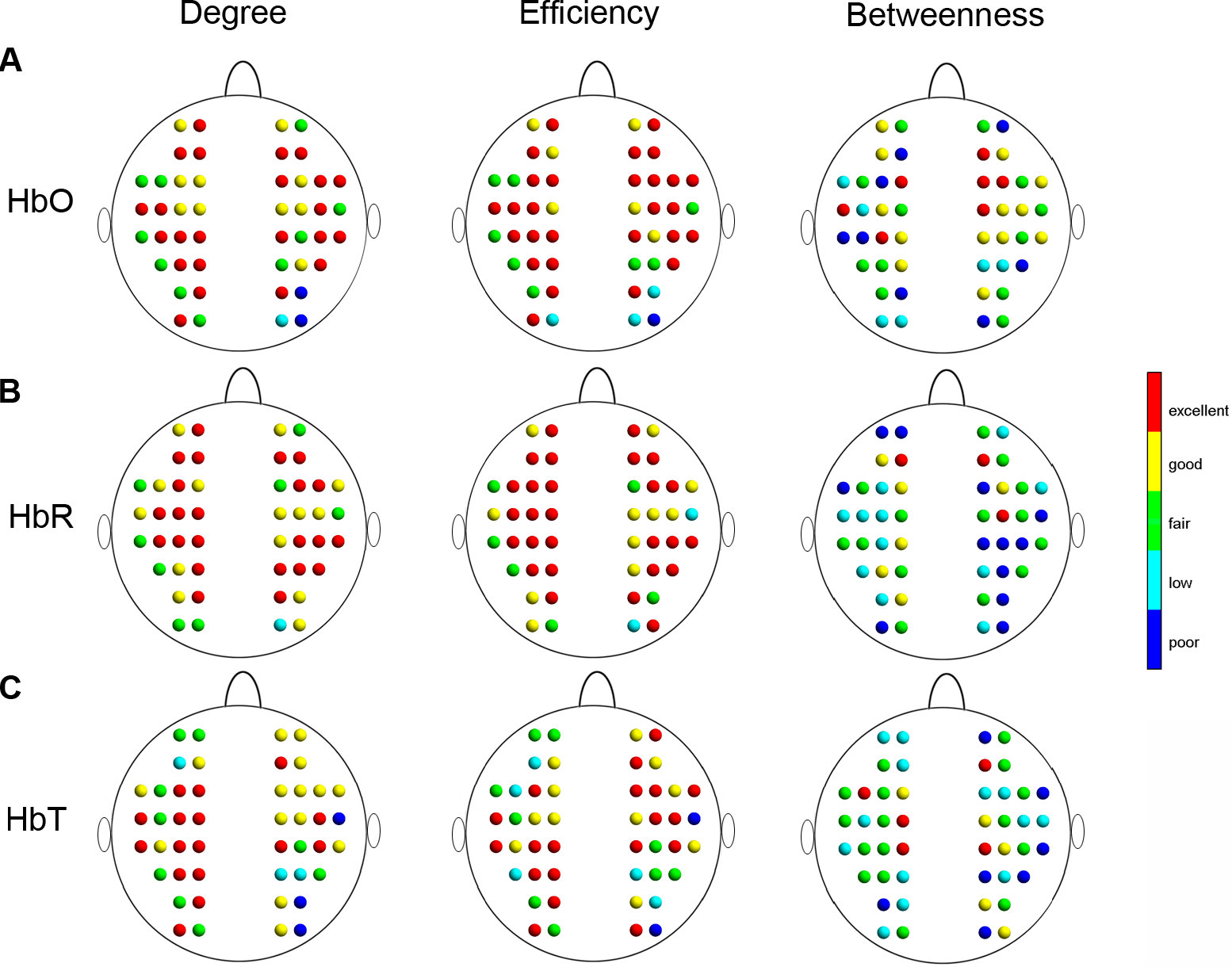


**Figure S5.** Threshold-independent reliability analysis of ICA-derived nodal centrality metrics. The areas under the curves (AUCs) of each nodal metric were used to provide threshold-independent reliability evaluation. (A-C) The nodal reliability was derived from HbO, HbR, and HbT, respectively. Different colors in the nodes correspond to different reliability grades: red, yellow, green, cyan, and blue colors represent excellent ( 0.75< *ICC* <1), good (0.6 < *ICC* < 0.75), fair (0.4 < *ICC* < 0.6), low (0.25 < *ICC* < 0.4), and poor (*ICC* < 0.25) reliability of the nodal centrality metrics, respectively.
